# Candidate Genes That May Be Responsible for the Unusual Resistances Exhibited by *Bacillus pumilus* SAFR-032 Spores

**DOI:** 10.1371/journal.pone.0066012

**Published:** 2013-06-14

**Authors:** Madhan R. Tirumalai, Rajat Rastogi, Nader Zamani, Elisha O’Bryant Williams, Shamail Allen, Fatma Diouf, Sharon Kwende, George M. Weinstock, Kasthuri J. Venkateswaran, George E. Fox

**Affiliations:** 1 Department of Biology and Biochemistry, University of Houston, Houston, Texas, United States of America; 2 Department of Biology, Texas Southern University, Houston, Texas, United States of America; 3 The Genome Institute, Washington University School of Medicine, St. Louis, Missouri, United States of America; 4 Biotechnology & Planetary Protection Group, NASA Jet Propulsion Laboratories, California Institute of Technology, Pasadena, California, United States of America; University of Connecticut, United States of America

## Abstract

The spores of several *Bacillus* species, including *Bacillus pumilus* SAFR-032 and *B. safensis* FO-36b, which were isolated from the spacecraft assembly facility at NASA's Jet Propulsion Laboratory, are unusually resistant to UV radiation and hydrogen peroxide. In order to identify candidate genes that might be associated with these resistances, the whole genome of *B. pumilus* SAFR-032, and the draft genome of *B. safensis* FO-36b were compared in detail with the very closely related type strain *B. pumilus* ATCC7061^T^. 170 genes are considered characteristic of SAFR-032, because they are absent from both FO-36b and ATCC7061^T^. Forty of these SAFR-032 characteristic genes are entirely unique open reading frames. In addition, four genes are unique to the genomes of the resistant SAFR-032 and FO-36b. Fifty three genes involved in spore coat formation, regulation and germination, DNA repair, and peroxide resistance, are missing from all three genomes. The vast majority of these are cleanly deleted from their usual genomic context without any obvious replacement. Several DNA repair and peroxide resistance genes earlier reported to be unique to SAFR-032 are in fact shared with ATCC7061^T^ and no longer considered to be promising candidates for association with the elevated resistances. Instead, several SAFR-032 characteristic genes were identified, which along with one or more of the unique SAFR-032 genes may be responsible for the elevated resistances. These new candidates include five genes associated with DNA repair, namely, BPUM_0608 a helicase, BPUM_0652 an ATP binding protein, BPUM_0653 an endonuclease, BPUM_0656 a DNA cytosine-5- methyltransferase, and BPUM_3674 a DNA helicase. Three of these candidate genes are in immediate proximity of two conserved hypothetical proteins, BPUM_0654 and BPUM_0655 that are also absent from both FO-36b and ATCC7061^T^. This cluster of five genes is considered to be an especially promising target for future experimental work.

## Introduction

The resistance of bacterial endospores to various chemical and physical agents/treatments poses a major challenge in combating contamination [1], [2]. The 1967 Outer Space Treaty requires that harmful contamination of celestial bodies, including Mars, that might harbor life be avoided [3]. As a result, planetary protection requirements for space flight missions involved with life detection strictly stipulate the sterilization/decontamination of spacecraft equipment [4], [5], [6], [7], [8]. Despite the maintenance of stringent nutrient-limiting, oligotrophic conditions, filtered air circulation, controlled temperature, humidity, and the use of a chemical disinfectant, bacterial populations producing unusually resistant spores of several *Bacillus* sp., have been isolated from spacecraft assembly facilities [9], [10], [11], [12]. Organisms producing such spores are not only more likely to survive the rigors of interplanetary transfer [13], [14], [15], [16], but could also prove to be repositories of evolving genes that could be transferred to pathogenic *Bacillus* strains [17], [18]. Amongst the *Bacillus* species isolated from spacecraft assembly facilities, it has been reported that *B. pumilus* SAFR-032 was able to tolerate nearly all environmental stresses, including growth at high temperature (65°C), high-alkalinity (pH 11), space vacuum, and simulated Mars environmental conditions [16], [19], including high levels of perchlorate [20]. *B. pumilus* SAFR-032 spores, exhibit significantly elevated resistance to both UV radiation and H_2_O_2._ The spores of another strain isolated from the same econiche, *B. safensis* FO-36b, also exhibits elevated resistances that exceed those of most other endospore producing strains [21], [22].

In fact, the levels of resistance seen in these two organisms far exceed those of the *B. subtilis* type strain [11], [22], which is used as a dosimetric strain [20]. The UV resistance levels of SAFR-032 [11] are comparable to that of species in the genus *Deinococcus* [23]. In order to begin to understand the basis of these elevated resistances, the genome of SAFR-032 was previously sequenced and genes involved in DNA repair, recombination, and peroxide resistance were compared to those found in *B. subtilis* and *B. licheniformis* [24].

This prior study is herein extended to a comparison between the genomes of SAFR-032, the type strain, *B. pumilus* ATCC7061^T^ and *B. safiensis* FO-36b. SAFR-032 is much more closely related to the ATCC7061^T^ strain, than it is to either *B. subtilis* or *B. licheniformis* [21]. Based on 16S rRNA and *gyrB* sequence comparisons, *B. safiensis* is in a clade immediately adjacent to that containing the *B. pumilus* strains [21]. Despite its close relationship to SAFR-032 and *B. safiensis*, the ATCC7061^T^ strain produces spores that don’t exhibit elevated resistance to either UV or H_2_O_2_ [9], [22].

Previous genome comparisons of non-sporulating radiation resistant bacteria yielded significant insight to the origins of resistance [25]. For example, a global comparison of the genomes of *Deinococcus geothermalis*, *D. radiodurans*, *Kineococcus radiotolerans*, and *Rubrobacter xylanophilus* indicated that all the basal DNA repair genes exhibited positive Darwinian selection [25]. Under exposure to ionizing radiation, a small subset of *Deinococcus* genus-specific genes including a novel class of single-stranded DNA binding protein coding genes have been shown to be up regulated as well as to play a role in genome reconstitution in [26]. Specific genes, such as *ygjD*, *yeaZ* and *recF*, though not unique to *Deinococcus sp*. have been identified as playing roles in DNA repair [27], [28]. In contrast, in the case of *Thermococcus gammatolerans* the high radio-resistance is probably due to proteins that remain to be characterized rather than a large arsenal of known DNA repair enzymes [29].

Herein, a detailed genomic comparison between two strains, *B. pumilus* SAFR-032 and *B. safensis* FO-36b, that producing highly resistant spores and a very closely related strain *B. pumilus* ATCC7061^T^ that does not produce resistant spores is undertaken. The objective is to identify possible genes and other genomic features that may be responsible for the changes in resistance. It is not expected that a clear explanation can be had from genome comparison alone. However, if a modest list of likely candidates can be identified, this will facilitate future experiments such as the construction of knockout mutants for candidate genes.

## Materials and Methods

### Genome Sequences

The whole genome sequence of SAFR-032 (Refseq accession no: NC_009848.1) and the draft genome of ATCC7061^T^ (Refseq accession no: NZ_ABRX00000000.1), consisting of 16 contigs were obtained from the public databases of the National Center for Biotechnology Information (NCBI). The ATCC7061 contigs were mapped against the SAFR-032 genome using the Projector software [30] resulting in an essentially contiguous sequence with two small gaps (Figure S1). In addition, preliminary sequence data consisting of 408 contigs of *B. safensis* FO-36b was obtained from the Baylor College of Medicine Human Genome Sequencing Center website at http://www.hgsc.bcm.tmc.edu. The online features/tools of the J Craig Venter Institute’s Comprehensive Microbial Resource [31] were used to quantify the GC content.

### Genome Comparison

Individual gene sequences from the SAFR-032 genome and the ATCC7061^T^ draft genome were blasted against the entire other genome as well as the available F-036b sequence data using the standalone version of NCBI’s BLAST program [32]. Since the FO-36b genome sequence was available only as numerous small individual contigs, at times only qualitative estimations of the presence/absence of a gene could be made. In particular, the genome location and conservation of the immediate surrounding neighborhood frequently could not be ascertained for FO-036b.

Genes with BLAST results in which the best hit had an e-value greater than (an arbitrary) 0.001 were considered absent from the target genome, while those with BLAST e-values below e-20 were considered to be matches. Genes with e-values between e-20 and 0.001 were further analyzed by aligning the sequence of the entire gene neighborhood with the corresponding region in the other genomes to ascertain/verify the BLAST results as well as to look for unusual features in the sequence.

### Sequence Divergence

The divergence levels of genes were quantified in the form of the amino acid/protein identity percentage with their homologs. These values were calculated using the identity similarity matrix in Bioedit (http://www.mbio.ncsu.edu/BioEdit/bioedit.html) or PSI-Blast [33]. The genome display tool (GDT) [34] and the Joint Genome Institute’s Integrated Microbial Genomes database and comparative analysis system’s ortholog neighborhood feature were used to visualize various findings.

Bioedit or MEGA [35] were used to obtain multiple sequence alignments. In some cases, individual genes in known operons showed significant differences in the degree of divergence from other genes in the same operon when SAFR-032 was compared to one or both of the other organisms. To understand this better, the homologs from the two most closely related organisms, typically *B. subtilis* and *B. licheniformis* were examined. When a *B. subtilis/B.licheniformis* homolog was not available, the next closest homolog was included in the identity percentage calculations.

### Prediction of Probable Protein Function(s)

Online protein domain annotation tools such as SMART (version 7) [36], InterProScan [37], SignalP 4.0 [38] and PSORT-B [39] were used to predict the probable cellular localization of genes of unknown function. The tool SecretomeP (version 2.0) [40] was used to further identify non-classically secreted proteins (proteins lacking signal peptides).

## Results

### Genomic Features

The circular chromosome of SAFR-032 (3,704,465 bases) has 3825 genes and is slightly smaller than that of *B. subtilis* (4,214,630 nt) and *B. licheniformis* (4,222,597 nt). The SAFR-032 genome in fact shares substantial colinearity with *B. subtilis* (Figure S2) as well as *B. licheniformis* (Figure S3). Moreover, the majority of the SAFR-032 protein-encoding sequences are also found in *B. subtilis* and *B. licheniformis.* The available coverage of the FO-36b genome is insufficient to allow assembly of the genome, but does allow an assessment of whether or not homologs of various genes are present. The salient features of these three genomes as compared with other *Bacillus* genomes were tabulated (Table S1).

### Classification of Genes

Genes that are unique to SAFR-032 or possibly uniquely shared with FO-36b are most likely to be associated with the unusual resistance properties exhibited by SAFR-032 spores. A detailed comparison of genes in SAFR-032 relative to ATCC7061^T^and FO-36b specifically, and other organisms in general, was undertaken. The genes were classified by their likely function. Genes of unknown function were considered to encode hypothetical proteins, (HP), when no clear homolog was found in other organisms. If a homolog was found, the genes were designated as encoding conserved hypothetical proteins (CHP). When homologous CHP genes are found in many organisms, they usually encode a protein of unknown function.

SAFR-032 genes that lacked a homolog in either F0-36b or ATCC7061^T^or both were assigned to either of three categories. These were (1) genes present in SAFR-032 but absent in F0-36b and ATCC7061^T^, (2) genes shared between SAFR-032 and F036b, and (3) genes shared between SAFR-032 and ATCC7061^T^. While conducting these comparisons, the *cotD* and *cotG* homologs of SAFR-032 of ATCC7061^T^ that were missed in the earlier annotations were identified.

### Unique SAFR-032 Genes

The first category consists of 34 unique hypothetical (ORFs) genes which do not have any homolog in other bacteria (Table S2). Four additional genes are also considered to be unique to SAFR-032. BPUM_0558 (encoding a hypothetical protein) has 29% sequence identity with its nearest ortholog *ycf1*, which is a plastid/chloroplast gene coded as an uncharacterized protein (RefSeq no: YP_004891372.1) in the eukaryote, *Cephalotaxus wilsoniana*. No significant bacterial homolog was detected. BPUM_1645 has 41% homology with the *B. subtilis* gene *yjcP*. However, *yjcQ,* which forms an operon with *yjcP* [41], is missing from the SAFR-032 genome. Given this and the low sequence similarity, BPUM_1645 is treated here as being unique to the SAFR-032 genome. BPUM_1649 was previously misannotated as *yobJ* and instead is unique to SAFR-032 having just 23% identity with its nearest homolog. Finally, BPUM_1731 (encoding a flavin reductase) shares 83% sequence identity with the putative uncharacterized protein, BATDEDRAFT_15142 from the eukaryote *Batrachochytrium dendrobatidis* JAM81 with no significant bacterial hit and hence is also classified as unique to SAFR-032. Finally, two extra copies of the flagellin gene (BPUM_1151–1152) are also unique to SAFR-032. Thus, a total of 40 SAFR-032 unique open reading frames were identified (Table S2). With the exception of one cluster of four adjoining genes, these are distributed throughout the genome (Figure S4).

### Highly Characteristic SAFR-032 Genes

There are 130 SAFR-032 genes absent from both F-036b and ATCC7061^T^, but with homologs in other *Bacillus*/non-*Bacillus sp*. These are classified as SAFR-032 characteristic genes (Table S3). Of these genes, 57 share less than 50% identity with their nearest homologs in other *Bacillus*/non-*Bacillus* sp (Figure S5). The relative location of these genes in the SAFR-032 genome is shown in Figure S4. All of the 40 SAFR-032 unique (Table S2) as well as the 130 SAFR-032 characteristic genes (Table S3) classified under Category 1, were examined in detail to better understand the extent to which they were actually missing from ATCC7061^T^ and FO-36b. In 136 cases the missing gene is essentially completely deleted from ATCC7061^T^ and FO-36b while the same flanking regions/genes seen in SAFR-032 are still present in the other two genomes. In none of these cases, was a “replacement” gene found in the same context. In twelve cases, a portion of the open reading frame is still present without in-frame stop codons in either ATCC7061^T^or FO-36b. Eight of these genes encode hypothetical proteins in SAFR-032, with the remaining four associated with specific functions. In one additional case, the homolog of BPUM_1763 in the FO-36b genome is similarly abbreviated, apparently because, it terminates a contig. This gene was therefore not considered to be unique to SAFR-032, but rather shared between SAFR-032 and FO-36b.

Finally, there were 22 genes in which similar sequences are found at the expected location in either ATCC7061^T^ or FO36b or both. However, they are not annotated as coding regions in those organisms for various reasons. This frequently is because they have multiple in frame stop codons, thereby making them likely pseudogenes. In seven cases, there is only a single base deletion or insertion that results in-frame stop codons. In one case (BPUM_2970), the corresponding locations in FO-36b or ATCC7061^T^ show patches of significant similarity with SAFR-032, but both FO-36b and ATCC7061^T^ lack an ORF.

### Genes Shared by the Resistant Spore Producing SAFR-032 and FO-36b, but Missing in ATCC7061^T^

A total of 67 genes were found to be shared by SAFR-032 and FO-36b with no annotated homolog in ATCC7061^T^ (Figure S6 red blocks). Thirteen of these have less than 50% similarity with their nearest homologs in other *Bacillus*/non-*Bacillus* sp, while four don’t have any detectable homolog at all in other species or strains (Figure S6, green blocks). There are two large gene clusters, one of which includes the operons *cgeCDE, cgeAB* and *msrB (yppQ)-msrA (yppP)*. This second category is summarized in Table S4.

Another 105 genes are shared by SAFR-032 and ATCC7061^T^, while annotated as absent in FO-36b. Thirty one of these genes have less than 50% identity, with the nearest 10 homologs found in other species. Sixteen genes shared by SAFR-032 and ATCC7061^T^ in fact lack obvious homologs in other genomes. Because the FO-36b genome is incomplete, it is possible that some of the genes in this category are in fact present in all three organisms (Table S5).

### Domain Analysis

Of the 37 SAFR-032 unique hypothetical proteins, 18 were predicted to be possible membrane proteins, one a possible transcriptional regulator, while no functional/localization domain(s) could be predicted for the remaining 18 proteins. Interestingly, 20 of these 37 proteins were predicted to be non-classically secreted.

### Pseudogenes

In addition to the unique and pairwise shared genes described above, there are also a number of pseudogenes of similar distribution. As listed in Table S6, there are 34 examples in SAFR-032 that appear to be pseudogenes due to base deletions, insertions, or premature in frame stop codons. All of these examples have intact homologs in either ATCC7061^T^or FO36b, or both. If there is a homolog in only one of the other genomes, homologs were sought and found in other *Bacillus* species. In addition, there are twelve genes that are intact in either ATCC7061^T^or FO-36b, but appear to be pseudogenes in the other two organisms due to base deletions/insertions. The three genomes share six pseudogenes that have intact homologs/ORFs in other *Bacillus*/non-*Bacillus* species. Several of the putative SAFR-032 pseudogenes are involved in functions that appear significant for cell survival. These include genes encoding ribosomal initiation factor IF3, ribosomal protein S2, and the Rho transcriptional terminator. In each of these cases, there is a single insertion or deletion that disrupts the correct reading frame. This suggests these may actually be functional genes that only appear to be pseudogenes/frameshifts because of minor genomic sequencing errors. However, we have mapped short Illumina reads to the genome on several occasions for other purposes and have not seen evidence of sequencing errors. It is therefore more likely that the single insertion/deletion events may be tolerated due to translational hopping [42], [43] or some similar phenomenon.

## Discussion

### Candidate Genes that may be Associated with Elevated Resistance

Spore DNA is preserved and protected from radiation and oxidative damage by the combined actions of several enzymes, spore-specific DNA binding (small acid soluble) proteins (SASPs), the spore-specific dipicolinic acid (DPA) and the intricate spore coat network, all of which are regulated through pathways governed by several hundred genes [44], [45], [46], [47], [48], [49]. Further, it has been shown recently that in *B. subtilis*, the homologous recombination (HR) and non-homologous end joining (NHEJ) DNA repair pathways are needed for spore survival under proton radiation [50].

The increased resistances seen in SAFR-032 and FO-36b spores may also be associated with changes in the complex network of genes associated with sporulation, spore coat formation, and spore germination [51]. Spore germination genes are also important because organism survival depends on the ability to recover from the sporulation stage despite potentially significant genomic damage. A number of genes from these functional categories are in fact missing from SAFR-032 (Table 1), when compared to *B. subtilis*. However, in each case, these genes are also missing in ATCC7061^T^ and FO-36b and therefore their absence is not likely to be associated with elevated spore resistance.

**Table 1:** Genes uniquely missing in the genomes of *B. pumilus* SAFR-032, *B. pumilus* ATCC7061^T^ & *B. safensis* FO-36B.

Gene Category		Gene name
Spore coat		*cotF, yraD*, *yraE*, *yraF*, *yraG, cotJA-JB-JC* (*cotJC* is also a peroxide resistance gene), *pbpE-racX* operon, *sspH & csgA* (SASP genes), *ydhD, yisY, ywqH, ypz, sunA, yrbC*
Spore regulation		*paiAB, rapB, rapC, rapF, rapG, rapI, rapJ* & their cognate pair regulatory genes, *phrC, phrF, phrG, phrI* & *phrK, sipV*
DNA repair	Class U Class V Class G & O	*uvrX, yjhB dinB mutT, yxlJ* (*aag*)
Peroxide resistance	Class G & O Class V+G & O Class V otheroxidative stress related genes	*ahpCF, ydbD katA, katB(katE) mrgA ylaABCD*
Germination		*yndDEF, yfkQRT*

Some of the 40 SAFR-032 unique open reading frames (Table S2) may encode proteins that have replaced the functions of one or more of the missing genes and as a result contribute to the observed elevated spore resistance. However, none of these genes were found in replacement contexts. Whereas the missing proteins are typically larger than 100 residues, most of the SAFR-032 unique open reading frames would encode proteins of less than 100 amino acid residues. It is unlikely that such small peptides would play a major role in conferring resistance. Nevertheless, in the absent of experimental studies it is not possible to readily assess the significance of most members of the list. In contrast, genes that are absent from ATCC7061^T^, but have distant relatives in other organisms, are potentially more promising because something may be known about their possible function. There are 130 of these SAFR-032 characteristic genes (Table S3) and several are known to be associated with relevant processes. In particular, there are five SAFR-032 characteristic genes that are involved in DNA repair processes in other organisms. These include BPUM_0608 a helicase, BPUM_0652 an ATP binding protein, BPUM_0653 an endonuclease, BPUM_0656 a DNA cytosine methyltransferase (EC 2.1.1.37), and BPUM_3674 a DNA helicase. In addition to being absent from both both ATCC7061^T^ and FO-36b, four of these genes are also missing in *B. subtilis* and *B. lichenformis* and have only 21–52% protein sequence identity with their most similar orthologs.

Three of these putative DNA repair genes, BPUM_652, 653, and 656, are in close proximity in the genome where they are separated by BPUM_654 and 655. These latter two genes are missing from ATCC7061^T^ and are also highly characteristic of SAFR-032 while encoding putative proteins of 342 and 589 residues. A detailed examination of this genome region reveals that BPUM_650 through BPUM_655 are all encoded on the same strand and thus may be part of an operon. In addition, as annotated, the coding regions of BPUM_652–656 are all partially overlapping as may occur when there is translational coupling.

The spores of both SAFR-032 and FO-36b exhibit elevated resistances. Therefore, genes uniquely shared by them are also of potential interest. There are four hypothetical proteins (BPUM_0757, 1124, 3099 and 1638) (Figure S6 and Table S4) that are uniquely shared by SAFR-032 and FO-36b. One of these, BPUM_0757, encodes a 171 residue protein, whereas the others would encode very small proteins of only 50–62 residues. It should be noted that the resistance properties of these two organisms differ [9] and hence the adaptations that lead to resistance are likely at least in part different. Thus, a single shared gene is unlikely to be solely responsible.

Instead of adding or deleting genes, enhanced resistance may have been obtained by modification of existing genes. Genes that have undergone recent selection frequently exhibit higher levels of sequence variation than genes that have not [52]. Thus, extremely rapid change can be an indicator of adaptation among universal genes. The levels of sequence (protein/amino acid sequence) similarity between homologs in the five gene groups listed in Table 1 were examined. In order to understand how much sequence variation was unusual, a comparison of 121 genes present in both *B. subtilis* and SAFR-032 in the five functional categories of interest was undertaken. The average similarity was 74.7% +/−10.8.

The α/β-type SASP, intact spore coat layers, reduced spore water content, DPA and spore pigmentation are the most important factors in determining spore survival and protection from mutagenic damage under simulated Mars conditions [49], [50]. All of the genes shown to be involved in these processes share >80% protein sequence identity between the three genomes as well as with the next nearest *Bacillus* relative. A number of spore coat proteins do exhibit rapid change. However, the SAFR-032 spores are beige in color, and thus lack the significant pigmentation that would likely be present if these proteins were involved in the enhanced resistance. Detailed discussion of these spore coat protein genes is provided in association with Table S7.

### Peroxide Resistance

Although radiation resistance has been the primary focus in this study, SAFR-032 and FO-36b spores also exhibit elevated resistance to peroxide. Two peroxide resistance genes, *yjqC* (BPUM_2346, encoding a spore manganese catalase) and, *ydbD* (BPUM_1305, encoding a manganese (Mn) catalase), have been proposed to function synergistically with other spore coat oxidoreductases to contribute to the enhanced peroxide resistance of SAFR-032 spores [53]. BPUM_1305 is shared by all three genomes, however, consistent with this proposal, BPUM_2346 is absent in the non-resistant ATCC7061^T^. In addition, SAFR-032 and FO-36b uniquely share two genes, BPUM_1716, which encodes a NADH-dependent flavin oxidoreductase and BPUM_1721 that encodes a flavin reductase.

### Assessment of Previously Identified Candidate Genes

The previous analysis [24] suggested that several key differences between SAFR-032 genes and their homologs from *B. subtilis* and *B. licheniformis*, might reflect adaptation to UV. The SAFR-032 DNA repair enzyme, Ada (BPUM_1200), was cited as a possible candidate because of its large amount of sequence change relative to *B. subtilis* and *B. licheniformis* homologs. However, BPUM_1200 is shared with both FO-36b and ATCC7061^T^ without unusual levels of sequence variation. Another possible contributor to UV resistance noted previously was the photolyase enzyme PhrB (BPUM_1378) that is absent from both *B. subtilis* and *B. licheniformis*. PhrB has been reported to be involved in the overall protection against UV irradiation through either enzymatic photoreactivation as in the unicellular photosynthetic cyanobacterium *Synechocystis sp*. PCC 6803 [54], or by enabling proper DNA supercoiling as in *Neisseria gonorrhoeae* [55]. In *E. coli*, the DNA photolyase monomerizes cyclobutane dimers in DNA back to the nucleotides [56]. However, this gene has not been shown to be involved in spore protection, but only in vegetative cells [56]. *phrB* is found in both FO-36b and ATCC7061^T^ without unusual levels of sequence variation. Likewise, *splB,* which encodes a spore photoproduct lyase and its regulatory gene *splA* and eight peroxide resistance genes indicated as possibly contributing to the H_2_O_2_ resistance of SAFR-032 in the previous report, are also shared by FO-36b and ATCC7061^T^. Thus, it is unlikely that any of these previous ten candidate genes are contributing to the enhanced UV/peroxide resistance of *B. pumilus* SAFR-032 (Table S8).

### Other Aspects of the SAFR-032 Genome

The transcriptional regulator Spx (BPUM_1077) regulates the oxidative stress resistance genes *msrB (yppQ)-msrA (yppP).* These genes encode peptide methionine sulfoxide reductases important for the regeneration of methionine and restoration of protein function after oxidative damage [57]. While the gene for Spx (BPUM_1077) is present in the three genomes examined here, its target genes *msrB (yppQ)-msrA (yppP)* (BPUM_1900–1901) are missing from the non-resistant ATCC7061^T^ genome (Tables S4, S8).

Gene duplications in prokaryotes have often been linked to environmental adaptation [58]. SAFR-032 possesses five copies of the flagellin gene (BPUM_0150, BPUM_1149–1152), of which, two (BPUM_1151–1152) are unique to SAFR-032 (Table S2). Another two BPUM_1149 and 1150 are uniquely shared with ATCC7061^T^, and one, BPUM_0150, is absent from both ATCC7061^T^ and FO-36b but shared by other *Bacillus* sp.

Another feature of the SAFR-032 genome is the occurrence of the *spoIIIC* as both a separate gene and as part of the composite gene *sigK* (containing the *spoIIIC* and *spoIVCB* halves fused together). *spoIIIC* encodes the C-terminal half of the mother-cell RNA polymerase sigma-factor gene *sigK*, while *spoIVCB* encodes the N-terminal half [59]. The presence of both versions (*spoIIIC* and *sigK*) in the SAFR-032 genome may be a consequence of where the cells were on the growth curve when their DNA was extracted. These genes were previously misannotated in SAFR-032 as *spoIIIC* and a pseudogene of *sigK*, respectively. The corrected annotation for these genes is: *sigK* (BPUM_2309) and *spoIIIC* (BPUM_2315).

### Conclusions

The genomic comparison undertaken here utilized much more closely related genomes than were previously available [24], with the result that all the genes previously listed as promising candidates for association with the elevated resistances can now likely be excluded because they are present in ATCC7061^T^ and do not exhibit the unusual levels of sequence variation that would be indicative of recent adaptation. In their place, a new set of candidate genes has been identified. By necessity, this list must include the forty open reading frames that are completely unique to SAFR-032 and four genes that are uniquely shared with FO-36b. It is noteworthy that many of these open reading frames are less than 150 residues in length. Since they are unique, by definition it is not clear if proteins are actually being produced and if they are, what their function might be, though many may be membrane proteins.

Of special interest are the five putative DNA repair genes that are absent from ATCC7061^T^ as well as *B. subtilis* and *B. lichenformis*. Three of these genes are in immmediate proximity of two SAFR-032 characteristic genes of unknown function that encode conserved hypothetical proteins of 342 and 589 amino acids that have orthologs in other organisms. Together this group of five genes represent an especially promising target for future experimental studies.

In summary, the detailed comparison of the SAFR-032, FO-36b and ATCC7061^T^ genomes presented here reveals several possibilities of genes that may be associated with the differences in resistance seen in the spores of these organisms [9], [11], [19]. Indeed, it is likely that many genes are actually involved to differing extents. The goal here was to narrow the possibilities and seek to identify candidates that may play a major role. To this end, the results provide several promising targets for future experimental work in which the candidate genes can be simply knocked out, or moved to a genome of a strain that lacks the resistance.

## Supporting Information

Figure S1 ATCC7061^T^ contigs mapped against SAFR-032 genome; the gaps are encircled.
(TIF)

Figure S2 Colinearity graph of *B. subtilis* and SAFR-032 genomes.
(TIF)

Figure S3 Colinearity graph of *B. licheniformis* and SAFR-032 genomes.
(TIF)

Figure S4 Genomic location of SAFR-032 unique genes. The *B. pumilus* SAFR-032 genome is represented as a series of small boxes that preserve their order of occurrence using the genome display tool [34]. Each box represents a single gene with progression being horizontal from left to right. Thus, genes 1 to 60 are in the first row, 61–120 in the second row etc. All SAFR-032 unique genes are filled in black.
(TIF)

Figure S5 Genomic location of SAFR-032 genes not shared by either ATCC7061^T^, or, FO-36B. The *B. pumilus* SAFR-032 genome is represented as a series of small boxes that preserve their order of occurrence using the Genome Display Tool [34]. Each box represents a single gene with progression being horizontal from left to right. Thus, genes 1 to 60 are in the first row, 61–120 in the second row etc. All SAFR-032 genes belonging to Category One, and not shared by either ATCC7061^T^ or F-036b are colored in green, red, or blue. The green blocks represent SAFR-032 unique genes. Blue blocks represent SAFR-032 genes in which the nearest homolog has less than 50% sequence identity. The Red boxes are the remaining category 1 SAFR-032 genes.
(TIF)

Figure S6 Genes uniquely shared between SAFR-032 and FO-36b. Genes that are shared by SAFR-032 and FO-36b with homologs in others *Bacillus* strains but not in ATCC7061^T^ are highlighted in red. Four genes that are completely unique to SAFR-032 and FO-36b are shown in green. There are two large clusters of shared genes.
(TIF)

Table S1 Salient features of the SAFR-032 and ATCC7061^T^ genomes as compared with other *Bacillus* species.
(DOCX)

Table S2 SFR-032 unique genes.
(DOCX)

Table S3 SAFR-032 characteristic genes.
(DOCX)

Table S4 Genes Shared by SAFR-032 and FO-36b but absent in ATCC7061^T^.
(DOCX)

Table S5 Genes Shared by SAFR-032 andATCC7061^T^ but absent in FO-36b.
(DOCX)

Table S6 Putative pseudogenes.
(DOCX)

Table S7 Conserved SAFR-032 genes exhibiting unusual sequence divergence.
(DOCX)

Table S8 List of DNA repair and peroxide resistance genes and their presence/absence in the three genomes.
(DOCX)
