# Supplementary figures and images for: Candidate Genes That May Be Responsible for the Unusual Resistances Exhibited by Bacillus pumilus SAFR-032 Spores
Source: PLoS One. 2013 Jun 14;8(6):e66012. doi: 10.1371/journal.pone.0066012 (PMC3682946; doi:10.1371/journal.pone.0066012)

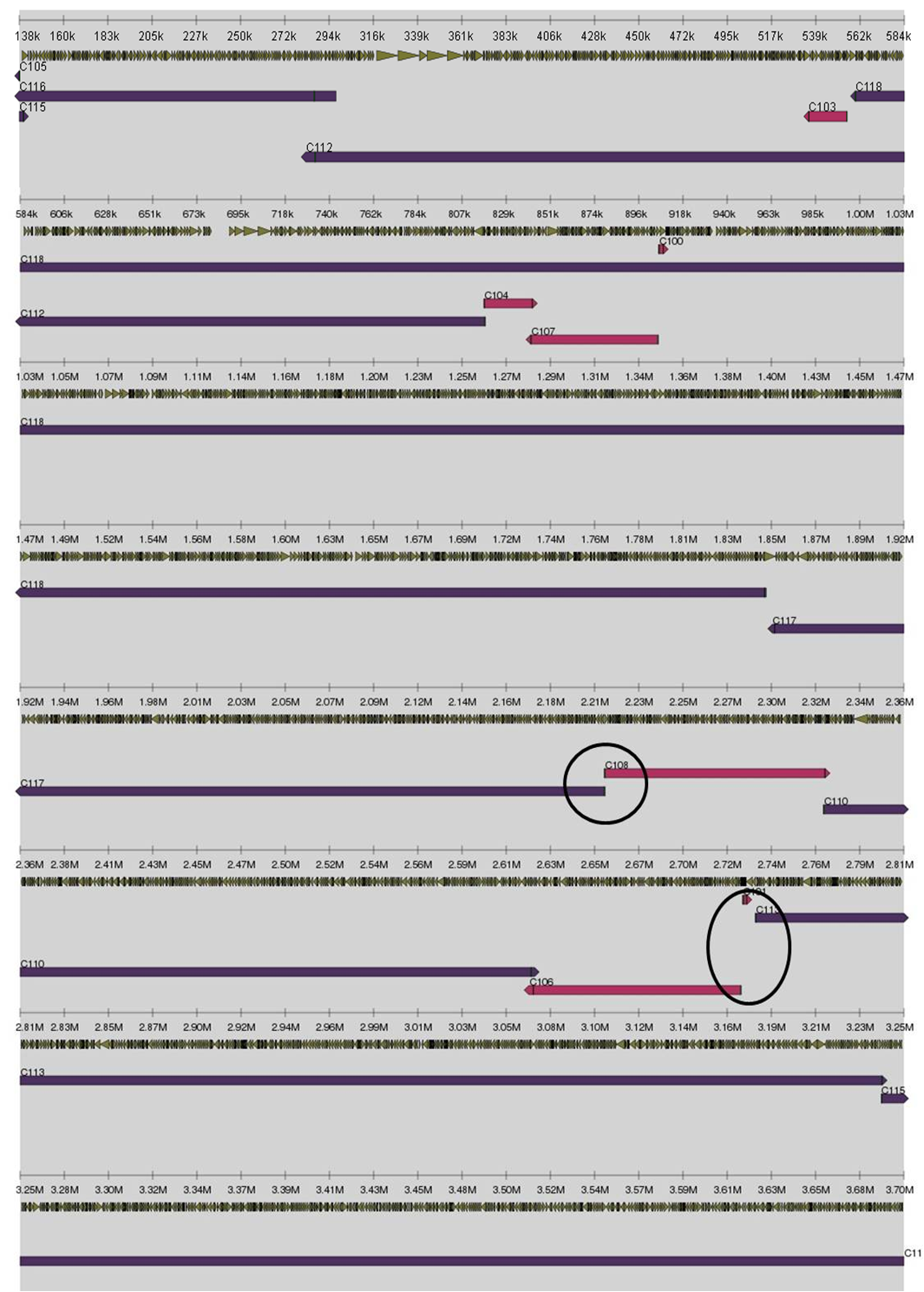

Supplement: Figure S1 — ATCC7061T contigs mapped against SAFR-032 genome; the gaps are encircled. (TIF) [file pone.0066012.s001.tif]

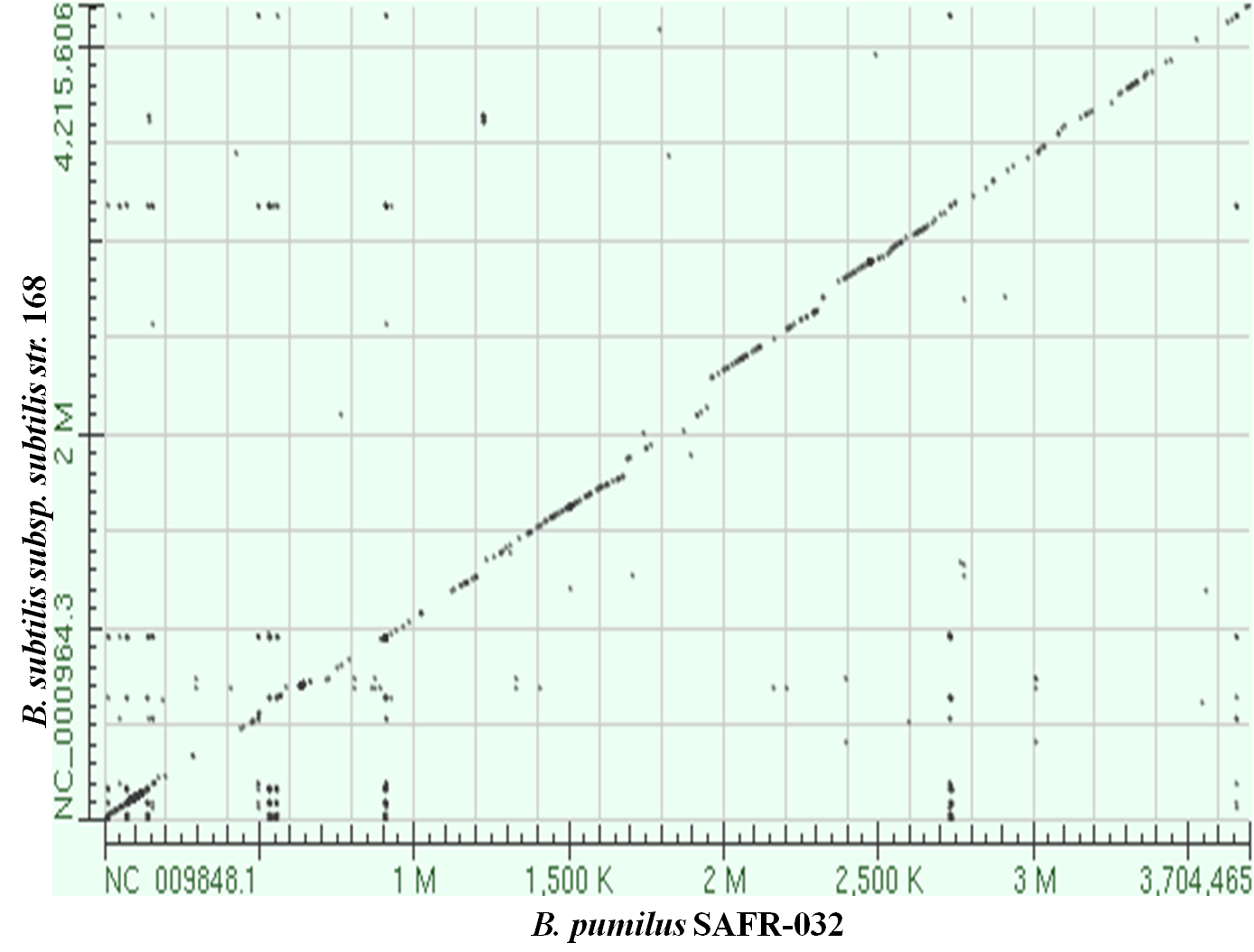

Supplement: Figure S2 — Colinearity graph of B. subtilis and SAFR-032 genomes. (TIF) [file pone.0066012.s002.tif]

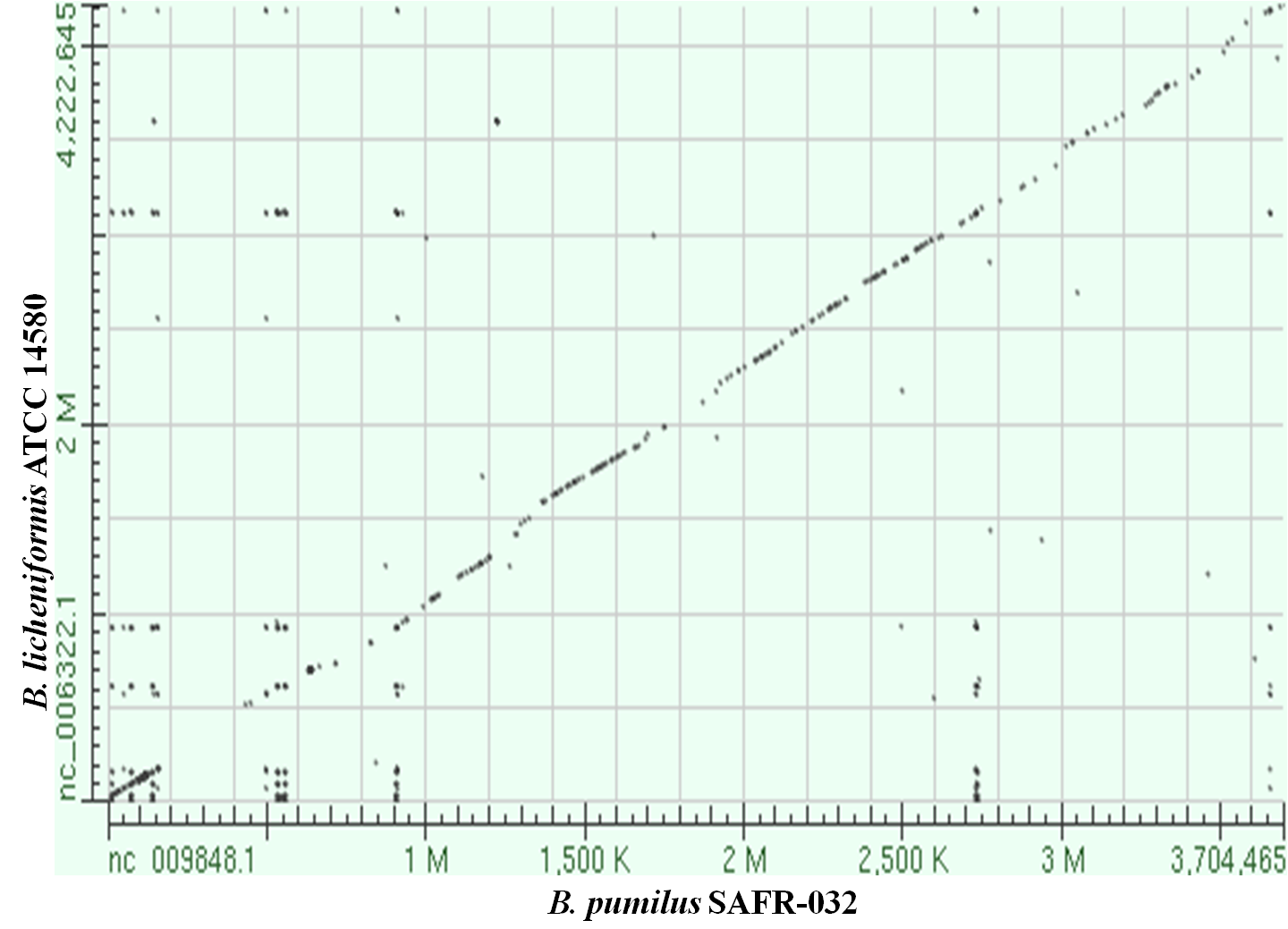

Supplement: Figure S3 — Colinearity graph of B. licheniformis and SAFR-032 genomes. (TIF) [file pone.0066012.s003.tif]

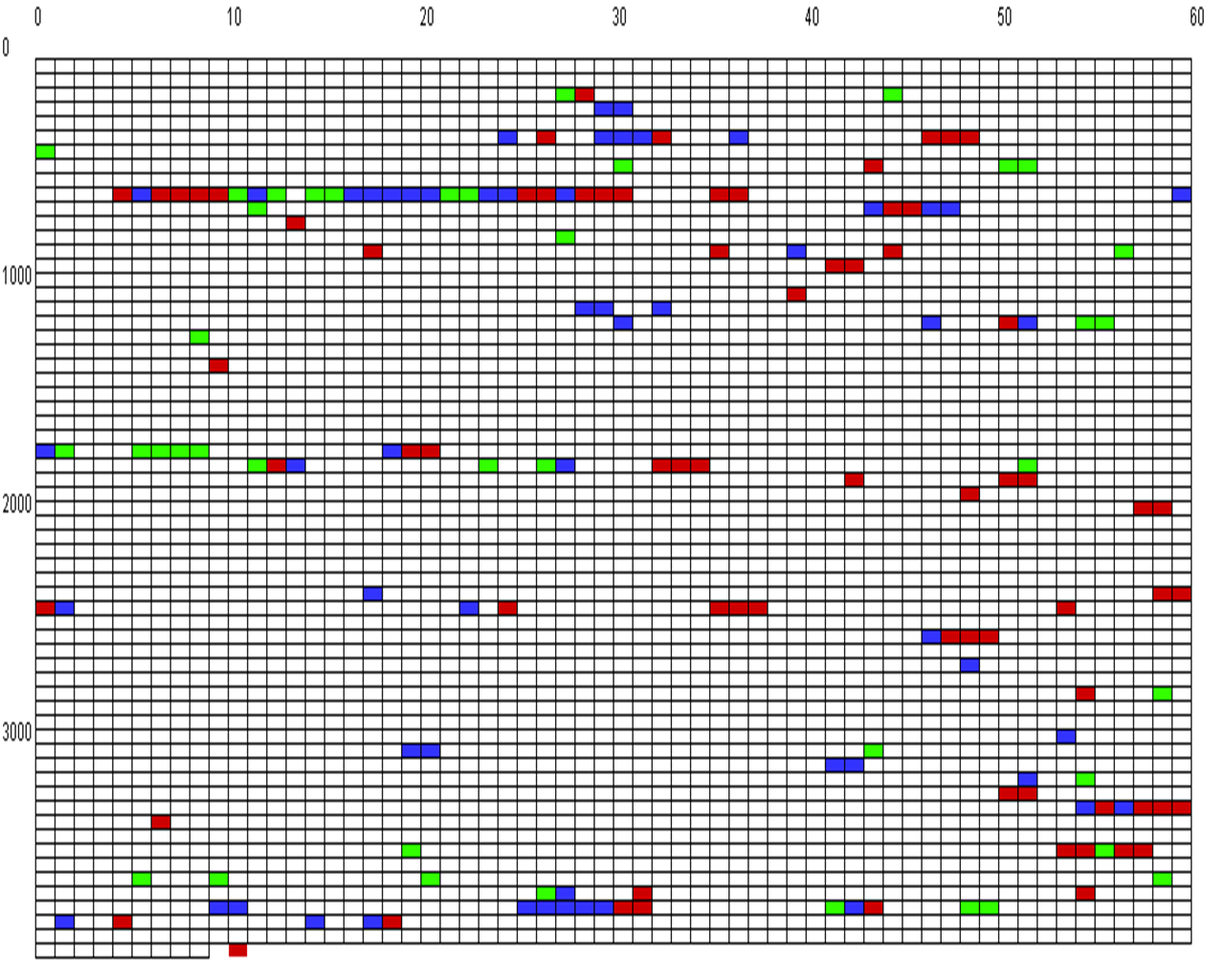

Supplement: Figure S5 — Genomic location of SAFR-032 genes not shared by either ATCC7061T, or, FO-36B. The B. pumilus SAFR-032 genome is represented as a series of small boxes that preserve their order of occurrence using the Genome Display Tool [34]. Each box represents a single gene with progression being horizontal from left to right. Thus, genes 1 to 60 are in the first row, 61–120 in the second row etc. All SAFR-032 genes belonging to Category One, and not shared by either ATCC7061T or F-036b are colored in green, red, or blue. The green blocks represent SAFR-032 unique genes. Blue blocks represent SAFR-032 genes in which the nearest homolog has less than 50% sequence identity. The Red boxes are the remaining category 1 SAFR-032 genes. (TIF) [file pone.0066012.s005.tif]

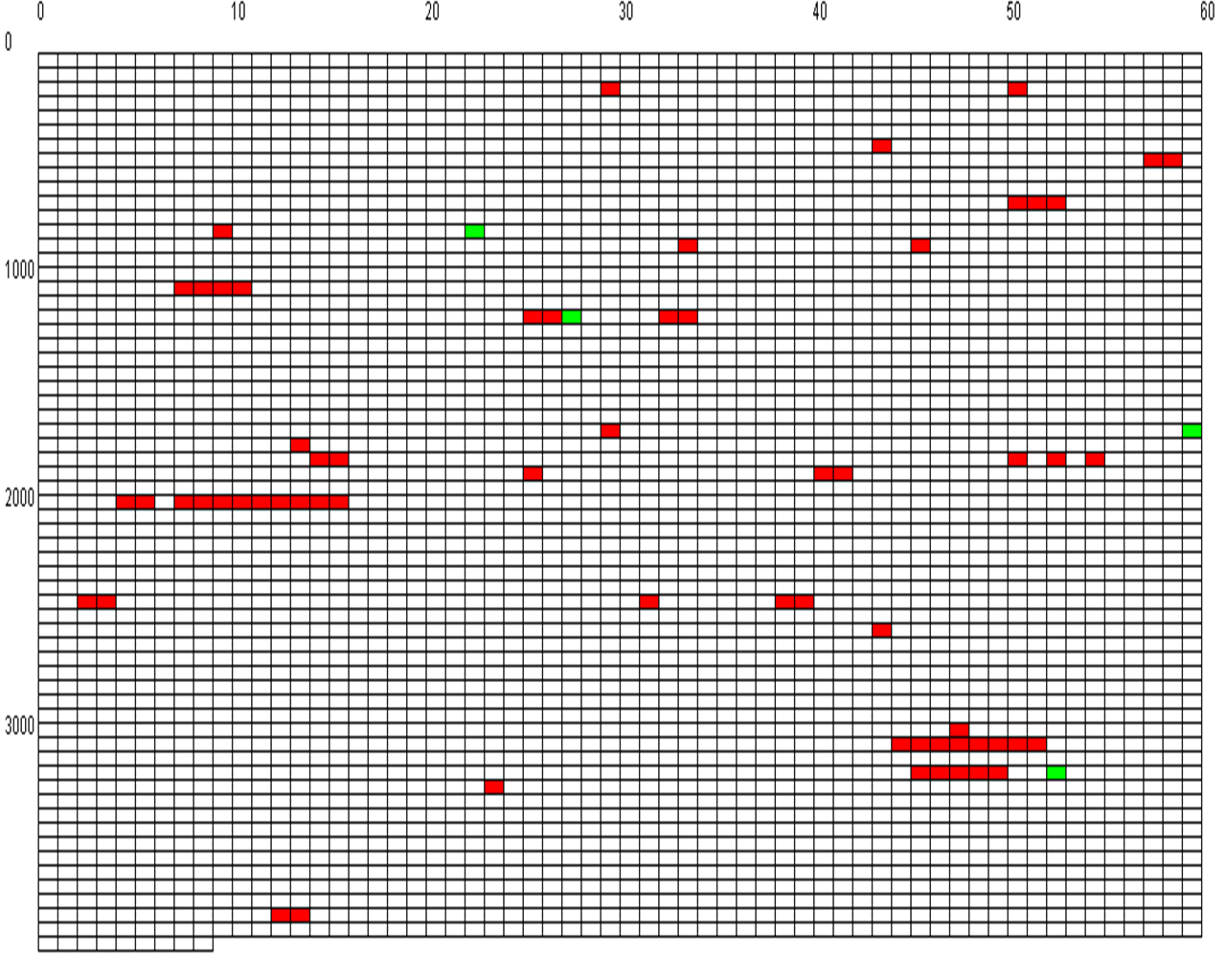

Supplement: Figure S6 — Genes uniquely shared between SAFR-032 and FO-36b. Genes that are shared by SAFR-032 and FO-36b with homologs in others Bacillus strains but not in ATCC7061T are highlighted in red. Four genes that are completely unique to SAFR-032 and FO-36b are shown in green. There are two large clusters of shared genes. (TIF) [file pone.0066012.s006.tif]
