# Supplementary material for: Candidate Genes That May Be Responsible for the Unusual Resistances Exhibited by Bacillus pumilus SAFR-032 Spores
Source: PLoS One. 2013 Jun 14;8(6):e66012. doi: 10.1371/journal.pone.0066012 (PMC3682946; doi:10.1371/journal.pone.0066012)
Supplement: Table S1 — Salient features of the SAFR-032 and ATCC7061T genomes as compared with other Bacillus species. (DOCX) [file pone.0066012.s007.docx]

**Table S1: Salient features of SAFR-032 and ATCC7061 genomes compared with other *Bacillus* genomes**

|  | ***B. pumilus*** | | ***B. subtilis subsp. subtilis str* 168** | ***B. licheniformis*** | ***B. amyloliquifaciens*** |
| --- | --- | --- | --- | --- | --- |
|  | **SAFR-032** | **ATCC7061** |  |  |  |
| **Chromosome/genome length** | **3704465** | **3833998** | **4214630** | **4222748** | **3918589** |
| **G+C content** | **41%** | **41%** | **43%** | **46%** | **46%** |
| **No of protein-coding ORFs** | **3681** | **3899** | **4105** | **4196** | **3693** |
| **No. of RNA-coding ORFs** | **93** | **72** | **119** | **93** | **118** |
| **Total length of all ORFs** | **3259699** | **3277717** | **3732340** | **3719904** | **3506145** |
| **% genome covered by ORFs** | **88%** | **85%** | **88%** | **88%** | **89%** |
| **Ave length of ORFs** | **864** | **825** | **884** | **867** | **920** |
| **% of genes on the plus strand** | **49%** | **33%** | **48%** | **49%** | **49%** |
| **% of genes on the minus** | **51%** | **67%** | **52%** | **51%** | **51%** |
