# Supplementary material for: Candidate Genes That May Be Responsible for the Unusual Resistances Exhibited by Bacillus pumilus SAFR-032 Spores
Source: PLoS One. 2013 Jun 14;8(6):e66012. doi: 10.1371/journal.pone.0066012 (PMC3682946; doi:10.1371/journal.pone.0066012)
Supplement: Table S2 — SFR-032 unique genes. (DOCX) [file pone.0066012.s008.docx]

**Table S2 SAFR-032 unique genes**

| **Type 1** | **Gene name** | **Locus tag BPUM no.** | **Function^6^** | **Protein length** | **% identity** |
| --- | --- | --- | --- | --- | --- |
|  | **flavin reductase^1,2^** | **1731** | **DNA repair** | **186** | **83** |
|  | **flagellin *hag4*^3^** | **1151** | **Motility and sporulation** | **301** | **NH** |
|  | **flagellin *hag5*^3^** | **1152** | **Motility and sporulation** | **299** | **NH** |
|  | **Hypotheticals** | **476^*^** | **possible membrane protein** | **138** | **NH** |
|  |  | **477** | **possible transcriptional regulator** | **146** | **NH** |
|  |  | **558^2^** | **possible membrane protein** | **216** | **29** |
|  |  | **560^*^** | **possible membrane protein** | **274** | **NH** |
|  |  | **562** | **None** | **98** | **NH** |
|  |  | **563** | **None** | **93** | **NH** |
|  |  | **569^*^** | **possible membrane protein** | **55** | **NH** |
|  |  | **570^*^** | **possible membrane protein** | **82** | **NH** |
|  |  | **1640** | **None** | **192** | **NH** |
|  |  | **1645^4*^** | **possible membrane protein** | **160** | **41** |
|  |  | **1646^*^** | **None** | **68** | **NH** |
|  |  | **1649^5*^** | **possible membrane protein** | **298** | **23** |
|  |  | **1650** | **None** | **93** | **NH** |
|  |  | **1714** | **None** | **49** | **NH** |
|  |  | **1735^*^** | **possible membrane protein** | **53** | **NH** |
|  |  | **1760** | **None** | **74** | **NH** |
|  |  | **2743** | **None** | **36** | **NH** |
|  |  | **3404^*^** | **None** | **61** | **NH** |
|  |  | **3534^*^** | **possible membrane protein** | **51** | **NH** |
|  |  | **3563^*^** | **possible membrane protein** | **371** | **NH** |
|  |  | **3638^*^** | **possible membrane protein** | **64** | **NH** |

**Type 1: SAFR-032 characteristic genes completely deleted while the same flanking regions/genes seen in SAFR-032 are still present in FO-36b and ATCC-7061; % identity - with the nearest homolog (from PSI-BLAST results); CHP – Conserved hypothetical protein; HP – Hypothetical protein; NH = No homolog (SAFR-032 unique genes); NA = not applicable;**

*** predicted to be possible non-classically secreted proteins;**

**1 - genes were reported in the previous study[10]; their uniqueness to SAFR-032 is confirmed with their absence from the two closest relatives of SAFR-032, namely the JPL isolate *Bacillus safensis* F036B and the *Bacillus pumilus* type strain ATCC-7061; 2 - the % identity is shared with eukaryotic genomes (*Batrachochytrium dendrobatidis* JAM81 & *Cephalotaxus wilsoniana*); 3 - extra gene copy; 4 – only one homolog in *B. subtilis yjcP* (only 41% identity); however, since *yjcQ* which forms an operon with *yjcP* is missing from the SAFR-032 genome, this is likely only a *yjcP* analog misannotated as *yjcP* – most likely unique to the SAFR-032 genome; 5 – misannotated as *yobJ*, likely unique to the SAFR-032 genome; 6 - prediction based on domain analysis**

| **Type 2** | **Locus tag (BPUM no)** | **Function** | **Protein length** | **ATCC-7061** | **F-036b** | **% identity** |
| --- | --- | --- | --- | --- | --- | --- |
|  | **762** | **None** | **51** | **A^1^** | **A^1^** | **NH** |
|  | **3480** | **None** | **51** | **A^1^** | **A^1^** | **NH** |
|  | **3645^*^** | **possible membrane protein** | **321** | **A^1^** | **A^1^** | **NH** |
|  | **3646^*^** | **possible membrane protein** | **297** | **A^1^** | **A^1^** | **NH** |

**Type 2: genes in which a portion of the open reading frame is present without stop codons in either FO-36b or ATCC-7061 (A^1^); in the case of FO-36b these genes may be partial because they terminate a contig; A = completely absent;**

**Function predicted based on domain analysis.**

| **Type 3** | **Locus Tag (BPUM no)** | **Function** | **Protein length** | **ATCC-7061** | **F-036b** | **% identity** |
| --- | --- | --- | --- | --- | --- | --- |
|  | **149** | **None** | **64** | **A^1^** | **A^1^** | **NH** |
|  | **166^*^** | **possible membrane protein** | **65** | **Ps** | **Ps** | **NH** |
|  | **366^*^** | **possible membrane protein** | **51** | **A^2^** | **A^2^** | **NH** |
|  | **456^*^** | **possible membrane protein** | **53** | **A^2^** | **A^2^** | **NH** |
|  | **620^*^** | **None** | **73** | **Ps** | **Ps** | **NH** |
|  | **853^*^** | **None** | **49** | **A^2^** | **A^2^** | **NH** |
|  | **1165** | **None** | **68** | **A^1^** | **A** | **NH** |
|  | **2970** | **None** | **40** | **A^3^** | **A^3^** | **NH** |
|  | **3101** | **None** | **72** | **Ps** | **Ps** | **NH** |
|  | **3368** | **None** | **51** | **Ps** | **A** | **NH** |
|  | **3476^*^** | **possible membrane protein** | **81** | **A^2^** | **A** | **NH** |
|  | **3493** | **possible membrane protein** | **57** | **A^2^** | **A^2^** | **NH** |

**Type 3: genes with homologs in either FO-36b or ATCC-7061, or both, with stop codons**

**Ps = pseudogene; A^1^ = sequence analog without an ORF;**

**A^2^ = corresponding homolog has base deletion(s)/insertions, causing in-frame stop codons, possibly due to sequencing errors;**

**A^3^ = gene with the corresponding locations in FO-36b or ATCC-7061 showing patches of significant similarity with SAFR-032, but both lacking an ORF; 4 = extra gene copy.**
