# Supplementary material for: Candidate Genes That May Be Responsible for the Unusual Resistances Exhibited by Bacillus pumilus SAFR-032 Spores
Source: PLoS One. 2013 Jun 14;8(6):e66012. doi: 10.1371/journal.pone.0066012 (PMC3682946; doi:10.1371/journal.pone.0066012)
Supplement: Table S3 — SAFR-032 characteristic genes. (DOCX) [file pone.0066012.s009.docx]

**Table S3 SAFR-032 characteristic genes**

| **Type 1** | **Gene class** | **Name** | **Locus tag** | **Protein length** | **% identity** |
| --- | --- | --- | --- | --- | --- |
|  | **DNA repair** | **Helicase^1^** | **608** | **1015** | **25** |
|  |  | **ATP-binding protein** | **652** | **496** | **48** |
|  |  | **Endonuclease** | **653** | **936** | **52** |
|  |  | **DNA (cytosine-5-) methyltransferase^1^** | **656** | **548** | **43** |
|  |  | **DNA helicase** | **3674** | **815** | **45** |
|  | **Peroxide resistance** | **NADH-dependent flavin oxidoreductase^1^** | **1716** | **344** | **74** |
|  | **Sporulation** | **FtsK/SpoIIIE family protein YdcQ** | **554** | **444** | **63** |
|  |  | ***spoIIIC* (C-terminal half of *sigK*)^2^** | **2315** | **NA** | **NA** |
|  | **Structural integrity** | **C40 family peptidase YddH (CwlT)** | **576** | **325** | **71** |
|  |  | **cell wall-associated protein WapA** | **2339** | **2340** | **74** |
|  |  | **Lipoprotein** | **2314** | **219** | **37** |
|  | **Hypotheticals** | **CHP** | **211** | **245** | **29** |
|  |  | **CHP** | **212** | **105** | **44** |
|  |  | **CHP** | **334** | **128** | **35** |
|  |  | **CHP** | **335** | **326** | **29** |
|  |  | **CHP** | **552** | **80** | **54** |
|  |  | **CHP** | **553** | **321** | **35** |
|  |  | **CHP** | **556** | **75** | **60** |
|  |  | **CHP YddA** | **557** | **92** | **54** |
|  |  | **CHP** | **559** | **847** | **39** |
|  |  | **CHP** | **564** | **130** | **31** |
|  |  | **CHP** | **565** | **409** | **49** |
|  |  | **CHP** | **566** | **270** | **36** |
|  |  | **CHP** | **567** | **253** | **34** |
|  |  | **CHP** | **568** | **119** | **47** |
|  |  | **CHP YddB** | **571** | **331** | **48** |
|  |  | **CHP YddC** | **572** | **86** | **41** |
|  |  | **CHP YddD** | **573** | **155** | **61** |
|  |  | **CHP YddG** | **575** | **793** | **38** |
|  |  | **CHP YddI** | **577** | **163** | **50** |
|  |  | **CHP^3^** | **578** | **69** | **90** |
|  |  | **CHP^3^** | **654** | **342** | **83** |
|  |  | **CHP** | **655** | **589** | **49** |
|  |  | **CHP** | **1069** | **119** | **31** |
|  |  | **CHP** | **1127** | **131** | **36** |
|  |  | **CHP** | **1143** | **96** | **30** |
|  |  | **CHP** | **1661** | **114** | **56** |
|  |  | **CHP** | **2269** | **349** | **34** |
|  |  | **CHP** | **2312** | **171** | **64** |
|  |  | **CHP** | **2337** | **56** | **41** |
|  |  | **CHP** | **2485** | **111** | **66** |
|  |  | **CHP** | **2487** | **65** | **52** |
|  |  | **CHP** | **2919** | **667** | **32** |
|  |  | **CHP** | **2946** | **287** | **26** |
|  |  | **CHP** | **2947** | **87** | **29** |
|  |  | **CHP** | **3028** | **183** | **32** |
|  |  | **CHP** | **3029** | **276** | **24** |
|  |  | **CHP** | **3098** | **183** | **47** |
|  |  | **CHP^3^** | **3402** | **105** | **82** |
|  |  | **CHP** | **3403** | **91** | **54** |
|  |  | **CHP** | **3405** | **131** | **50** |
|  |  | **CHP** | **3406** | **108** | **51** |
|  |  | **CHP** | **3607** | **165** | **39** |
|  |  | **CHP^4^** | **3625** | **173** | **35** |
|  |  | **CHP** | **3626** | **174** | **28** |
|  |  | **CHP** | **3627** | **73** | **54** |
|  |  | **CHP** | **3628** | **94** | **64** |
|  |  | **CHP** | **3639** | **247** | **28** |
|  |  | **CHP^4,5^** | **3640** | **62** | **80** |
|  |  | **CHP** | **3658** | **204** | **47** |
|  |  | **CHP** | **3661** | **326** | **71** |
|  |  | **CHP** | **3671** | **88** | **48** |
|  |  | **CHP** | **3675** | **253** | **53** |
|  | **Transporters** | **ABC transporter ATP-binding protein** | **336** | **428** | **35** |
|  |  | **ABC transporter ATP-binding protein** | **337** | **224** | **50** |
|  |  | **DASS family divalent anion:sodium (Na+) symporter** | **684** | **471** | **72** |
|  |  | **PTS family glucose/glucoside (glc) porter component IIBCA^6^** | **1742** | **630** | **60** |
|  |  | **AGCS family alanine or glycine:sodium (Na+) or proton (H+) symporter** | **2351** | **483** | **64** |
|  |  | **ABC transporter ATP-binding protein MglA** | **3564** | **217** | **37** |
|  |  | **major facilitator transporter YdeG^3^** | **3591** | **423** | **78** |
|  |  | **ABC transporter ATP-binding protein** | **3622** | **306** | **29** |
|  |  | **ABC transporter ATP-binding protein** | **3623** | **297** | **43** |
|  | **Transcriptional regulator** | **transcriptional regulator YdcR** | **555** | **366** | **59** |
|  |  | **AraC family transcriptional regulator YddE** | **584** | **290** | **65** |
|  |  | **TetR family transcriptional regulator** | **1148** | **204** | **38** |
|  |  | **MarR family transcriptional regulator** | **1717** | **143** | **44** |
|  |  | **BglG family transcriptional antiterminator LicT^6^** | **1743** | **280** | **57** |
|  |  | **LysR family transcriptional regulator YwqM** | **2352** | **297** | **56** |
|  | **Signal transduction** | **response regulator^3^** | **353** | **227** | **75** |
|  |  | **sensor histidine kinase** | **354** | **475** | **58** |
|  |  | **response regulator** | **3624** | **116** | **47** |
|  | **Biochemical pathways** | **Acetyltransferase** | **585** | **191** | **75** |
|  |  | **Methyltransferase** | **1065** | **250** | **37** |
|  |  | **alcohol dehydrogenase** | **1147** | **370** | **69** |
|  |  | **patatin phospholipase** | **1639** | **355** | **46** |
|  |  | **O-methyltransferase^3^** | **1879** | **304** | **84** |
|  |  | **1,3-propanediol dehydrogenase DhaT** | **2350** | **387** | **64** |
|  |  | **dehydrogenase^3^** | **2488** | **326** | **94** |
|  |  | **acyltransferase YkrP** | **2739** | **356** | **57** |
|  |  | **glycosyltransferase^3^** | **3157** | **707** | **57** |
|  |  | **glycosyltransferase^3^** | **3158** | **330** | **79** |
|  |  | **glycosyltransferase** | **3222** | **610** | **36** |
|  |  | **aminotransferase** | **3223** | **392** | **55** |
|  |  | **carbamoylphosphate synthase large subunit^6^** | **3224** | **426** | **47** |
|  |  | **CDP-glycerol glycerophosphotransferase^6^** | **3225** | **667** | **50** |
|  |  | **dTDP-glucose 4,6-dehydratase RfbB** | **3226** | **353** | **68** |
|  |  | **glucose-1-phosphate thymidylyltransferase** | **3227** | **294** | **72** |
|  |  | **glycosyltransferase TagFA** | **3234** | **1204** | **62** |
|  | **Genome recombination** | **Transposase** | **352** | **451** | **84** |
|  |  | **Transposase** | **814** | **451** | **83** |
|  |  | **Transposase** | **841** | **477** | **84** |
|  |  | **transposase OrfX^3^** | **898** | **107** | **83** |
|  |  | **integrase^3^** | **899** | **269** | **85** |
|  |  | **Transposase** | **1287** | **451** | **84** |
|  |  | **Transposase** | **1820** | **451** | **84** |
|  |  | **Integrase** | **1948** | **266** | **91** |
|  |  | **Transposase** | **1949** | **110** | **97** |
|  |  | **Transposase** | **2310** | **451** | **84** |
|  |  | **Transposase** | **2368** | **451** | **83** |
|  |  | **Transposase** | **2484** | **513** | **48** |
|  |  | **Transposase** | **3568** | **451** | **83** |
|  | **Translation** | **ribosomal-protein-alanine N-acetyltransferase YjcK** | **1821** | **186** | **71** |

**Type 1: SAFR-032 characteristic genes completely deleted while the same flanking regions/genes seen in SAFR-032 are still present in FO-36b and ATCC-7061; % identity - with the nearest homolog (from PSI-BLAST results); CHP – Conserved hypothetical protein; HP – Hypothetical protein; NH = No homolog (SAFR-032 unique genes); NA = not applicable;**

**1 - genes were reported in the previous study[10]; their uniqueness to SAFR-032 is confirmed with their absence from the two closest relatives of SAFR-032, namely the JPL isolate *Bacillus safensis* F036B and the *Bacillus pumilus* type strain ATCC-7061; 2 - in SAFR-032, unfused *spoIIIC* occurs as a separate gene; the fused *sigK* containing *spoIVCB (N-terminal half of SigK)* and *spoIIIC (C-terminal half of SigK)* occur as a single fused gene, a feature shared by ATCC-7061, F-036b and several other *Bacillus* sp.; 3 - eleven type 1 genes share > 75% identity with their respective non FO-36b and ATCC-7061 homologs; 4 – one homolog only; 5 - shares homolog with another recently isolated *B. pumilus* strain; 6 - extra gene copy;**

| **Type 2** | **Name** | **Locus tag** | **Protein length** | **ATCC-7061** | **F-036b** | **% identity** |
| --- | --- | --- | --- | --- | --- | --- |
|  | **CHP** | **469** | **122** | **A^1^** | **A** | **76** |
|  | **CHP** | **832** | **144** | **A^1^** | **A** | **66** |
|  | **CHP** | **1662** | **345** | **A^1^** | **A** | **70** |
|  | **CHP** | **3606** | **160** | **A^1^** | **A** | **45** |
|  | **flagellin *hag1*** | **150** | **322** | **Ps** | **A^1^** | **81** |
|  | **YddE (ConE)** | **574** | **818** | **A** | **A^1^** | **68** |
|  | **MFS family major facilitator transporter^5^** | **1066** | **414** | **A^1^** | **A^1^** | **37** |
|  | **glycosyl hydrolase** | **1741** | **488** | **A^1^** | **A** | **97** |

**Type 2: genes in which a portion of the open reading frame is present without stop codons in either FO-36b or ATCC-7061 (A^1^); in the case of FO-36b these genes may be partial because they terminate a contig; A = completely absent; Ps = pseudogene; A^2^ = non-homologous sequence without an ORF; 3 = gene misannotated as ‘*yfiS*’**

| **Type 3** | **Name** | **Locus Tag** | **Protein length** | **ATCC-7061** | **F-036b** | **% identity** |
| --- | --- | --- | --- | --- | --- | --- |
|  | **CHP** | **328** | **196** | **A^1^** | **A** | **46** |
|  | **CHP** | **330** | **32** | **A^2^** | **A^2^** | **50** |
|  | **CHP** | **341** | **194** | **A** | **Ps** | **45** |
|  | **CHP** | **836** | **107** | **A^1^** | **Ps** | **40** |
|  | **CHP^5^** | **1016** | **91** | **A^2^** | **A** | **62** |
|  | **CHP** | **1660** | **222** | **Ps** | **A** | **40** |
|  | **CHP** | **1736** | **255** | **Ps** | **A** | **30** |
|  | **CHP** | **2313** | **67** | **A** | **Ps** | **54** |
|  | **CHP** | **2611** | **202** | **A** | **A^2^** | **37** |
|  | **transposase** | **1812** | **451** | **Ps** | **A** | **84** |

**Type 3: genes with homologs in either FO-36b or ATCC-7061, or both, with stop codons**

**Ps = pseudogene; A^1^ = sequence analog without an ORF; A^2^ = corresponding homolog has base deletion(s)/insertions, causing in-frame stop codons, possibly due to sequencing errors; A^3^ = gene with the corresponding locations in FO-36b or ATCC-7061 showing patches of significant similarity with SAFR-032, but both lacking an ORF; 4 = extra gene copy.**
