# Supplementary material for: Candidate Genes That May Be Responsible for the Unusual Resistances Exhibited by Bacillus pumilus SAFR-032 Spores
Source: PLoS One. 2013 Jun 14;8(6):e66012. doi: 10.1371/journal.pone.0066012 (PMC3682946; doi:10.1371/journal.pone.0066012)
Supplement: Table S4 — Genes Shared by SAFR-032 and FO-36b but absent in ATCC7061T. (DOCX) [file pone.0066012.s010.docx]

**Table S4: Genes shared by SAFR-032 and FO-36b but absent in ATCC-7061**

| **Gene type** | **Gene** | **Locus tag** | **Protein length** | **% identity** |
| --- | --- | --- | --- | --- |
| **Unique to SAFR-032 and F036b** | **CHP** | **757** | **170** | **NH** |
|  | **CHP** | **1124** | **62** | **NH** |
|  | **CHP^*^** | **3099** | **60** | **NH** |
|  | **hypothetical bacteriophage protein YqaO^1^** | **1638** | **50** | **NH** |
| **Hypotheticals** | **CHP^2^** | **172** | **65** | **57** |
|  | **CHP** | **409** | **159** | **23** |
|  | **CHP^3^** | **744** | **133** | **42** |
|  | **CHP^4^** | **830** | **151** | **48** |
|  | **CHP YrpD** | **842** | **250** | **64** |
|  | **CHP** | **1123** | **127** | **71** |
|  | **CHP** | **1129** | **139** | **62** |
|  | **CHP** | **1655** | **157** | **61** |
|  | **CHP** | **1759** | **146** | **33** |
|  | **CHP** | **1761** | **152** | **51** |
|  | **CHP^5^** | **1763** | **110** | **41** |
|  | **CHP** | **1903** | **86** | **74** |
|  | **CHP** | **1904** | **221** | **28** |
|  | **CHP YpmT** | **1905** | **65** | **56** |
|  | **CHP YpmS^2^** | **1906** | **188** | **61** |
|  | **CHP** | **2316** | **83** | **37** |
|  | **CHP^6^** | **2317** | **138** | **52** |
|  | **CHP** | **2354** | **187** | **82** |
|  | **CHP** | **2973** | **447** | **38** |
|  | **CHP** | **2975** | **391** | **69** |
|  | **CHP** | **3669** | **81** | **74** |
|  | **CHP** | **3670** | **181** | **56** |
| **Sporulation** | ***gerA* (spore germination)^7^** | **1794** | **497** | **89** |
|  | ***cgeA* (spore maturation)** | **1896** | **134** | **63** |
|  | ***cgeC* (spore maturation)** | **1895** | **137** | **50** |
|  | **spore manganese catalase YjqC** | **2346** | **276** | **81** |
| **Transport** | **SulP family sulfate permease^3^** | **151** | **480** | **72** |
|  | **PTS family porter component IIBC MalP** | **660** | **526** | **88** |
|  | **ABC transporter ATP-binding proteins YclNOPQ** | **984**  **985**  **986**  **987** | **316**  **316**  **252**  **314** | **77**  **72**  **65**  **69** |
|  | **multidrug ABC transporter ATP-binding protein** | **2353** | **572** | **91** |
|  | **SSS family solute/sodium (Na+) symporter** | **2976** | **468** | **55** |
|  | **ABC superfamily ATP binding cassette transporters** | **3092**  **3093**  **3094**  **3095**  **3096** | **257**  **272**  **297**  **310**  **544** | **51**  **49**  **67**  **80**  **67** |
| **Biochemical pathways** | **histone acetyltransferase HPA2-like protein** | **483** | **207** | **48** |
|  | **maltose-6'-phosphate glucosidase** | **661** | **442** | **90** |
|  | **acetylxylan esterase^3^** | **1130** | **276** | **78** |
|  | **nitrite reductase (NAD(P)H) small subunit NasE^3^,**  **large subunit NasD** | **1810**  **1811** | **107**  **806** | **66**  **71** |
|  | **N-acylneuraminate cytidylyltransferase NeuA^3^** | **1898** | **238** | **35** |
|  | **glyoxalase** | **2481** | **131** | **77** |
|  | **Hydrolase** | **2913** | **299** | **48** |
|  | **3-oxoacyl-[acyl-carrier-protein] reductase** | **2971** | **248** | **76** |
|  | **4-hydroxythreonine-4-phosphate dehydrogenase, PdxA** | **2972** | **341** | **70** |
|  | **alcohol dehydrogenase** | **2977** | **390** | **78** |
|  | **dihydrodipicolinate synthase DapA3** | **2978** | **299** | **74** |
| **Peroxide resistance** | **Flavodoxin** | **1721** | **193** | **53** |
|  | **methionine sulfoxide reductases**  **MsrB(YppQ)-MsrA(Ypp P)** | **1900**  **1901** | **143**  **178** | **79** |
|  | **Thioredoxin** | **3130** | **212** | **73** |
| **Transcription pathways / regulation** | **transcriptional regulator** | **484** | **183** | **54** |
|  | **transcriptional regulator GlvR** | **659** | **254** | **82** |
|  | **transcriptional regulator** | **1122** | **112** | **82** |
|  | **MarR family transcriptional regulator YdzF** | **1722** | **144** | **62** |
|  | **MarR family transcriptional regulator** | **1902** | **143** | **77** |
|  | **transcriptional regulator** | **2974** | **593** | **38** |
| **Structural integrity** | **capsular polysaccharide biosynthesis protein D, CapD** | **1899** | **342** | **65** |
| **DNA repair** | **DNA mismatch repair protein MutS1^2^** | **1608** | **858** | **79** |

**CHP – conserved hypothetical protein; NH – no homolog**

*** predicted to be non-classically secreted.**

**1 - Extra gene copy.**

**2 - The corresponding ATCC-7061 homolog has base deletion(s) causing in-frame stop codons, possibly due to sequencing error.**

**3 – A portion of the open reading frame is present without stop codons in ATCC-7061.**

**4 - In ATCC-7061, flanking genes terminate contigs.**

**5 - A portion of this gene terminates contig in F036B so inferred that it is likely a real gene.**

**6 - Only one non-SAFR-032/ FO-36b homolog - maybe unique to SAFR-032 & FO-36b.**

**7 –this is different from *gerAA-AB-AC* – *gerA* is a pseudogene in ATCC-7061, with chunks of the sequence deleted in the homolog.**
