# Supplementary material for: Candidate Genes That May Be Responsible for the Unusual Resistances Exhibited by Bacillus pumilus SAFR-032 Spores
Source: PLoS One. 2013 Jun 14;8(6):e66012. doi: 10.1371/journal.pone.0066012 (PMC3682946; doi:10.1371/journal.pone.0066012)
Supplement: Table S5 — Genes Shared by SAFR-032 andATCC7061T but absent in FO-36b. (DOCX) [file pone.0066012.s011.docx]

**Table S5: Genes shared by SAFR-032 and ATCC-7061, but absent in FO-36b.**

| **Unique to**  **SAFR-032**  **and ATCC-7061** | **Gene** | **Locus tag** | **Protein length** | **% identity** |
| --- | --- | --- | --- | --- |
|  | **CHP** | **178** | **99** | **NH** |
|  | **CHP** | **888** | **54** | **NH** |
|  | **CHP^1^** | **1299** | **58** | **NH** |
|  | **CHP** | **1919** | **24** | **NH** |
|  | **CHP** | **1947** | **70** | **NH** |
|  | **CHP** | **2482** | **59** | **NH** |
|  | **CHP** | **2875** | **138** | **NH** |
|  | **CHP^2^** | **3109** | **29** | **NH** |
|  | **CHP** | **3307** | **183** | **NH** |
|  | **CHP** | **3308** | **134** | **NH** |
|  | **CHP^1^** | **3313** | **59** | **NH** |
|  | **CHP** | **3366** | **58** | **NH** |
|  | **CHP** | **3609** | **136** | **NH** |
|  | **ECF family DNA-directed RNA polymerase sigma factor SigV^3^** | **588** | **181** | **NH** |
|  | **flagellin^4^ *hag2*** | **1149** | **300** | **NH** |
|  | **flagellin^4^ *hag3*** | **1150** | **305** | **NH** |
| **Hypotheticals** | **CHP^1^** | **359** | **124** | **45** |
|  | **CHP^2^** | **373**  **374**  **375** | **130**  **79**  **97** | **64**  **51**  **35** |
|  | **CHP** | **457** | **264** | **84** |
|  | **CHP** | **589** | **482** | **46** |
|  | **CHP** | **634** | **89** | **54** |
|  | **CHP** | **177** | **71** | **64** |
|  | **CHP** | **179** | **315** | **54** |
|  | **CHP** | **187** | **69** | **64** |
|  | **CHP** | **254** | **112** | **48** |
|  | **CHP** | **749** | **163** | **61** |
|  | **CHP** | **864** | **132** | **33** |
|  | **CHP** | **889** | **288** | **58** |
|  | **CHP** | **1128** | **62** | **75** |
|  | **CHP** | **1228** | **123** | **82** |
|  | **CHP** | **1311** | **140** | **41** |
|  | **CHP** | **1728** | **115** | **78** |
|  | **CHP** | **1732** | **303** | **66** |
|  | **CHP** | **1733** | **105** | **71** |
|  | **CHP** | **2110** | **90** | **71** |
|  | **CHP** | **2338** | **217** | **32** |
|  | **CHP** | **2483** | **62** | **90** |
|  | **CHP** | **2728** | **211** | **58** |
|  | **CHP** | **2746** | **57** | **57** |
|  | **CHP** | **2747** | **109** | **58** |
|  | **CHP** | **2748** | **292** | **68** |
|  | **CHP** | **2898** | **76** | **65** |
|  | **CHP** | **3100** | **101** | **82** |
|  | **CHP** | **3304** | **176** | **26** |
|  | **CHP** | **3305** | **185** | **31** |
|  | **CHP** | **3362** | **167** | **54** |
|  | **CHP** | **3363** | **204** | **44** |
|  | **CHP** | **3364** | **176** | **40** |
|  | **CHP** | **3365** | **377** | **54** |
|  | **CHP** | **3367** | **71** | **60** |
|  | **CHP** | **3369** | **63** | **51** |
|  | **CHP YozM** | **3382** | **92** | **39** |
|  | **CHP** | **3477** | **222** | **24** |
|  | **CHP** | **3481** | **112** | **46** |
|  | **CHP** | **3605** | **160** | **46** |
|  | **CHP** | **3608** | **496** | **24** |
|  | **CHP** | **3664** | **336** | **73** |
|  | **CHP** | **3665** | **703** | **43** |
|  | **CHP** | **3672** | **86** | **47** |
| **Transcription**  **regulation** | **LysR family transcriptional regulator** | **494** | **329** | **72** |
|  | **Xre family transcriptional regulator** | **550** | **128** | **40** |
|  | **LysR family transcriptional regulator** | **1227** | **287** | **85** |
|  | **MarR family transcriptional regulator** | **1729** | **141** | **77** |
|  | **TetR family transcriptional regulator** | **1730** | **189** | **62** |
|  | **transcriptional regulator** | **1740** | **132** | **78** |
|  | **transcriptional regulator** | **2598** | **148** | **43** |
|  | **TetR family transcriptional regulator** | **3694** | **203** | **53** |
| **sporulation** | **spore transcriptional regulator YopK^5^** | **549** | **372** | **36** |
|  | **FtsW/RodA/SpoVE family cell division protein** | **2729** | **384** | **74** |
| **DNA repair** | **DNA (cytosine-5-)-methyltransferase YdiP^5^** | **561** | **261** | **72** |
| **Transport** | **MFS family major facilitator transporter, macrolide:cation transporter YfmI** | **628** | **410** | **33** |
|  | **oligopeptide ABC transporter substrate-binding protein OppAB & OppF^4^** | **1070**  **1071**  **1074** | **552**  **309**  **309** | **48**  **70**  **84** |
|  |  |  |  |  |
|  | **MFS family major facilitator transporter** | **2065** | **419** | **68** |
|  | **ABC transporter ATP-binding prfotein YgaD** | **3306** | **603** | **41** |
|  | **MFS family major facilitator transporter** | **3417** | **386** | **55** |
| **Biochemical pathways** | **Metal-dependent hydrolase** | **139** | **248** | **79** |
|  | **aldo/keto dehydrogenase** | **495** | **262** | **85** |
|  | **thioesterase BacT** | **631** | **236** | **62** |
|  | **3-hydroxybutyryl-CoA dehydrogenase** | **632** | **285** | **56** |
|  | **methoxymalonyl-ACP biosynthesis protein** | **633** | **351** | **64** |
|  | **acyl-CoA dehydrogenase (NADP(+))** | **635** | **380** | **58** |
|  | **alkaline phosphatase PhoB** | **641** | **468** | **52** |
|  | **alanine racemase** | **979** | **368** | **70** |
|  | **acetyltransferase** | **1269** | **174** | **62** |
|  | **GNAT family acetyltransferase** | **1657** | **180** | **72** |
|  | **cytidine deaminase** | **1737** | **140** | **80** |
|  | **esterase^1^** | **1739** | **308** | **68** |
|  | **NAD(P)H dehydrogenase (quinone)^1^** | **2597** | **195** | **65** |
|  | **purine nucleosidase** | **3604** | **311** | **62** |
| **Competence** | **competence pheromone precursor ComX** | **2839** | **56** | **51** |
| **Other functions** | **glutamate rich protein GrpB^6^** | **458** | **168** | **64** |
|  | **cytochrome P450 protein** | **3695** | **401** | **65** |
| **Antibiotic resistance** | **nonribosomal peptide synthetase subunit** | **629** | **1470** | **40** |
|  | **beta-lactamase PbpX** | **630** | **513** | **39** |
|  | **nonribosomal peptide synthetase subunit** | **636** | **889** | **33** |
|  | **polyketide synthase subunit** | **637** | **1501** | **40** |
|  | **polyketide synthase subunit** | **638** | **2416** | **43** |
|  | **polyketide synthase subunit** | **639** | **2136** | **40** |
|  | **kanamycin kinase** | **1575** | **307** | **39** |
| **Translation** | **threonyl-tRNA synthetase ThrZ** | **761** | **643** | **70** |
| **Recombination** | **integrase YdcL** | **580** | **425** | **80** |

|  |  |  |
| --- | --- | --- |

**CHP – conserved hypothetical protein; NH – no homolog**

**1 - Portion of the open reading frame is present without stop codons in FO-36b;**

**2 - In FO36b, flanking genes terminate contigs;**

**3 - Extra gene copy;**

**4 - First half of the open reading frame is present without stop codons in FO-36b; second half is replete with base changes resulting in a pseudogene;**

**5 – A portion of the open reading frame is present without stop codons in ATCC-7061;**

**6 - Genome location not conserved.**
