# Supplementary material for: Candidate Genes That May Be Responsible for the Unusual Resistances Exhibited by Bacillus pumilus SAFR-032 Spores
Source: PLoS One. 2013 Jun 14;8(6):e66012. doi: 10.1371/journal.pone.0066012 (PMC3682946; doi:10.1371/journal.pone.0066012)
Supplement: Table S6 — Putative pseudogenes. (DOCX) [file pone.0066012.s012.docx]

**Table S6: Putative Pseudogenes**

| **Gene type** | **Function** | **Gene product** | **SAFR-032 Locus tag** |
| --- | --- | --- | --- |
| **Class A: Common to the 3 genomes** | **Hypotheticals** | **CHP** | **125** |
|  |  | **CHP** | **701** |
|  | **Other functions** | **possible NUDIX hydrolase** | **2318** |
|  | **translation** | **initiation factor IF-3** | **2532** |
|  | **transport** | **ABC superfamily ATP binding cassette transporter** | **3491** |
|  | **Structural integrity** | **cell surface protein** | **3528** |

| **Class B: Unique to SAFR-032 & ATCC-7061** | **Transcription regulation** | **transcriptional regulator ImmR** | **551** |
| --- | --- | --- | --- |
|  | **Hypotheticals** | **CHP** | **731** |
|  |  | **CHP** | **1734** |
|  |  | **CHP** | **3212** |
|  | **Other functions** | **TrkA domain-containing protein YrvC** | **2405** |
|  |  | **molybdopterin precursor biosynthesis protein (frameshift) MoaB^1^** | **2578** |
|  | **Cell division** | **FtsW/RodA/SpoVE family cell division protein (frameshift)^1^** | **2730** |

| **Class C: Unique to SAFR-032 & FO-36b** | **Translation** | **ribosomal protein S2 RpsB^1^** | **1548** |
| --- | --- | --- | --- |
|  | **Hypotheticals** | **CHP** | **1798** |
|  | **Transcription** | **ECF family DNA-directed RNA polymerase sigma subunit *xpf*** | **1641-43** |

| **Class D: Unique to SAFR-032** | **Hypotheticals** | **CHP YxkD^1^** | **225** |
| --- | --- | --- | --- |
|  |  | **CHP^2^** | **536** |
|  |  | **CHP^1^** | **778** |
|  |  | **CHP YdfS^1^** | **1678** |
|  |  | **HP^3^** | **2240** |
|  |  | **HP YrkO^4^** | **2920** |
|  |  | **CHP^1^** | **3409** |
|  | **Metabolic pathways** | **SAM-dependent methyltransferase YcgJ^1^** | **294** |
|  |  | **maltose O-acetyltransferase (*maa*)^1, 5^** | **1715** |
|  |  | **alcohol dehydrogenase** | **1718** |
|  |  | **endopeptidase LytF/YojL** | **1866** |
|  |  | **S8 family serine endopeptidase subtilisin SprE (frameshift) aprE2^1^** | **2086** |
|  |  | **aldehyde dehydrogenase (NAD(+)) frameshift YwdH^1^** | **3443** |
|  |  | **mannonate dehydratase UxuA^6^** | **3539** |
|  | **Transporters** | **APC family amino acid-polyamine-organocation transporter RocE** | **342** |
|  |  | **MFS family major facilitator transporter^7^** | **669** |
|  |  | **branched-chain amino acid transporter AzlC** | **1719** |
|  |  | **branched-chain amino acid transporter AzlD** | **1720** |
|  | **Recombination** | **integrase *int (ydcL)*** | **579** |
|  |  | **transposase (frameshift)^8^** | **2781** |
|  | **Structural integrity** | **collagen binding surface protein^4^** | **768** |
|  | **Other functions** | **MepB family protein^7^** | **1229** |
|  |  | **bipartite response regulator, C-terminal effector YqaQ^1, 9^** | **1644** |
|  |  | **internalin^3^** | **2239** |
|  |  | **fosfomycin resistance protein YndN (FosB)** | **3533** |
|  | **Phage proteins** | **terminase small subunit YqaS^10^** | **1647** |
|  |  | **possible hypothetical bacteriophage protein frameshift** **YqbB** | **1648** |
|  |  | **hypothetical bacteriophage protein** | **2486** |
|  | **Transcription regulation** | **transcriptional regulator^11^** | **1726** |
|  |  | **possible transcriptional regulator (frameshift) (YtcG) NrdR^1^** | **2544** |
|  |  | **transcription terminator Rho (frameshift)^12^** | **3351** |
|  | **Translation** | **histidine--tRNA ligase frameshift HisS** | **2397** |
|  |  | **cysteine desulfurase (NifS) IscS^1^** | **2428** |
|  |  | **cysteine desulfurase NifZ (frameshift)** | **2603** |

**1- single base deletion/addition/modification;**

**2- likely present in SAFR-032, with a base deletion only towards the last 10 bases of the gene;**

**3- the two genes BPUM_2239 and BPUM_2240 are two part pseudogenes of the intact internalin gene homolog in F-036b and is entirely absent in ATCC-7061.**

**4- SAFR-032 has the intact gene followed by its pseudogene;**

**5- corresponding intact gene/ORF (homolog) absent in ATCC-7061 only;**

**6- corresponding intact gene/ORF homolog entirely absent in F036-b, but segments present in ATCC-7061;**

**7- insertion of a small segment (3-4 bases);**

**8- corresponding genomic loci in both FO-36b and ATCC-7061 have neither the intact/complete homolog/ORF nor the pseudogene;**

**9- start codon misannotated in SAFR-032;**

**10- start codon & gene misannotated as *yjcR;***

**11- the complete gene (homolog)/ORF intact in ATCC-7061 only / absent in F036-b;**

**12- deletion/insertion of ≥2 base(s);**
