# Supplementary material for: Candidate Genes That May Be Responsible for the Unusual Resistances Exhibited by Bacillus pumilus SAFR-032 Spores
Source: PLoS One. 2013 Jun 14;8(6):e66012. doi: 10.1371/journal.pone.0066012 (PMC3682946; doi:10.1371/journal.pone.0066012)
Supplement: Table S7 — Conserved SAFR-032 genes exhibiting unusual sequence divergence. (DOCX) [file pone.0066012.s013.docx]

**Table S7 Conserved SAFR-032 genes exhibiting unusual sequence divergence(s)**

| **Gene type** | **Gene** | **Locus tag** | **Protein length** |
| --- | --- | --- | --- |
| **spore regulation** | **transcriptional regulator YopK^1^** | **549** | **372** |
| **DNA repair** | **DNA (cytosine-5-)-methyltransferase YdiP** | **561** | **261** |
| **sporulation related transporters** | **operon *oppABCDF*** | **1070-1074** | **552**  **309**  **343**  **354**  **309** |
| **spore coat formation** | ***cotG***^2^ | **NA** | **193** |
|  | ***cotZ**** | **1103** | **147** |
|  | ***cotY**** | **1105** | **163** |
|  | ***cotX**** | **1106** | **159** |
|  | ***cotW**** | **1107** | **127** |
|  | ***cotV**** | **1108** | **131** |
|  | ***cotD***^2^ | **NA** | **78** |

**1 - gene not found in FO-36b and shows substantial sequence divergence between SAFR-032 and ATCC-7061, as well as amongst other *Bacillus* sp;**

**2 - gene previously not annotated. Found in this study through sequence alignment. *cotD* codes for the ‘inner spore coat protein D’ and plays an important role in in spore coat structural integrity and thereby spore resistance properties[**[**57**](#_ENREF_57)**]. *cotG* codes for a morphogenetic protein that is required for maturation of the spore protein CotB[**[**58**](#_ENREF_58)**].**

**Percentage identity of protein products of SAFR-032 genes showing unusual sequence divergences with homologs from closely related species.**

|  | **cotD^2^** | | **cotV***** | | **cotW***** | | **cotX***** | | **cotY***** | | **cotZ***** | |
| --- | --- | --- | --- | --- | --- | --- | --- | --- | --- | --- | --- | --- |
|  | **α** | **β** | **α** | **β** | **α** | **β** | **α** | **β** | **α** | **β** | **α** | **β** |
| **SAFR-032 vs *B.subtilis*** | **64** | **67** | **53** | **40** | **48** | **32** | **57** | **45** | **77** | **86** | **71** | **75** |
| **SAFR-032 vs *B.licheniformis*** | **48** | **46** | **53** | **41** | **38** | **21** | **54** | **45** | **71** | **80** | **70** | **75** |
| **SAFR-032 vs ATCC-7061** | **97** | **99** | **93** | **93** | **60** | **48** | **68** | **64** | **96** | **99** | **96** | **97** |
| **SAFR-032 vs FO-36b** | **96** | **95** | **94** | **93** | **91** | **90** | **87** | **90** | **96** | **99** | **93** | **93** |
| **ATCC-7061 vs *B.subtilis*** | **64** | **66** | **56** | **42** | **47** | **31** | **54** | **44** | **77** | **86** | **71** | **75** |
| **ATCC-7061 vs *B.licheniformis*** | **47** | **45** | **55** | **39** | **39** | **25** | **53** | **42** | **70** | **80** | **68** | **74** |
| **ATCC-7061 vs FO-36b** | **94** | **94** | **94** | **92** | **61** | **52** | **64** | **62** | **98** | **100** | **95** | **94** |
| **FO-36b vs *B.subtilis*** | **64** | **64** | **54** | **40** | **46** | **30** | **57** | **44** | **77** | **86** | **71** | **73** |
| **FO-36b vs *B.licheniformis*** | **47** | **44** | **53** | **39** | **37** | **18** | **53** | **45** | **70** | **80** | **67** | **73** |
| ***B.subtilis* vs *B.licheniformis*** | **56** | **56** | **63** | **50** | **35** | **27** | **54** | **51** | **73** | **81** | **65** | **66** |

| ***cotG*^2^** | |  | ***ydiP*** | | ***yopK*** | |  |  |
| --- | --- | --- | --- | --- | --- | --- | --- | --- |
|  | **α** | **β** |  |  | **α** | **β** | **α** | **β** |
| **SAFR-032 vs *B.subtilis*** | **41** | **26** | **SAFR-032 vs *B.subtilis*** | **43** | **45** | **48** | **34** |  |
| **SAFR-032 vs *B.amyloliquifaciens*** | **39** | **24** | **SAFR-032 vs *B.amyloliquifaciens*** | **72** | **68** | **40** | **18** |  |
| **SAFR-032 vs ATCC-7061** | **77** | **78** | **SAFR-032 vs *B.anthracis*** | **66** | **53** | **45** | **21** |  |
| **SAFR-032 vs FO-36b** | **73** | **74** | **SAFR-032 vs ATCC-7061** | **43** | **39** | **48** | **29** |  |
| **ATCC-7061 vs *B.subtilis*** | **40** | **29** | **ATCC-7061 vs *B.subtilis*** | **71** | **64** | **53** | **42** |  |
| **ATCC-7061 vs *B.amyloliquifaciens*** | **38** | **26** | **ATCC-7061 vs *B.amyloliquifaciens*** | **44** | **43** | **35** | **17** |  |
| **ATCC-7061 vs FO-36b** | **88** | **89** | **ATCC-7061 vs *B.anthracis*** | **41** | **34** | **40** | **18** |  |
| **FO-36b vs *B.subtilis*** | **39** | **28** | ***B.subtilis* vs *B.amyloliquifaciens*** | **46** | **48** | **33** | **16** |  |
| **FO-36b vs *B.amyloliquifaciens*** | **37** | **27** | ***B.subtilis* vs *B.anthracis*** | **41** | **36** | **36** | **22** |  |
| ***B.subtilis* vs *B.amyloliquifaciens*** | **74** | **69** | ***B.amyloliquifaciens* vs *B.anthracis*** | **64** | **53** | **42** | **17** |  |

|  | **oppA^3^** | | **oppB^3^** | | **oppC^3^** | | **oppD^3^** | | **oppF^3^** | |
| --- | --- | --- | --- | --- | --- | --- | --- | --- | --- | --- |
|  | **α** | **β** | **α** | **β** | **α** | **β** | **α** | **β** | **α** | **β** |
| **SAFR-032 vs *B.subtilis*** | **46** | **28** | **52** | **53** | **51** | **44** | **66** | **69** | **69** | **80** |
| **SAFR-032 vs *B.licheniformis*** | **45** | **28** | **53** | **51** | **49** | **44** | **64** | **70** | **68** | **80** |
| **SAFR-032 vs ATCC-7061** | **48** | **31** | **52** | **48** | **50** | **44** | **67** | **69** | **84** | **89** |
| **SAFR-032 vs FO-36b** | **49** | **26** | **51** | **48** | **33** | **44** | **62** | **69** | **83** | **89** |
| **ATCC-7061 vs *B.subtilis*** | **70** | **73** | **73** | **89** | **74** | **81** | **76** | **85** | **72** | **84** |
| **ATCC-7061 vs *B.licheniformis*** | **68** | **67** | **67** | **72** | **67** | **70** | **73** | **85** | **71** | **83** |
| **ATCC-7061 vs FO-36b** | **97** | **77** | **96** | **97** | **60** | **99** | **87** | **98** | **93** | **99** |
| **FO-36b vs *B.subtilis*** | **71** | **57** | **72** | **87** | **48** | **82** | **71** | **85** | **72** | **84** |
| **FO-36b vs *B.licheniformis*** | **69** | **54** | **66** | **70** | **42** | **70** | **68** | **85** | **72** | **84** |
| ***B.subtilis* vs *B.licheniformis*** | **70** | **28** | **70** | **75** | **69** | **76** | **75** | **87** | **75** | **87** |

**α – DNA percentage identity; β – protein percentage identity**

| ***oppA*^3^** | | | ***oppB*^3^** | | | ***oppF*^3^** | | |  |
| --- | --- | --- | --- | --- | --- | --- | --- | --- | --- |
| **1^st^ segment** | **2^nd^ segment** | **Full length** | **1^st^ segment** | **2^nd^ segment** | **Full length** | **1^st^ segment** | **2^nd^ segment** | **Full length** |  |
| **SAFR-032 vs *B.subtilis*** | **47** | **45** | **46** | **54** | **52** | **52** | **62** | **71** | **69** |
| **SAFR-032 vs *B.licheniformis*** | **46** | **43** | **45** | **53** | **52** | **53** | **60** | **71** | **68** |
| **SAFR-032 vs ATCC-7061** | **48** | **47** | **48** | **52** | **52** | **52** | **64** | **92** | **84** |
| **SAFR-032 vs FO-36b** | **49** | **46** | **49** | **52** | **52** | **51** | **64** | **90** | **83** |
| **ATCC-7061 vs *B.subtilis*** | **70** | **72** | **70** | **79** | **72** | **73** | **73** | **72** | **72** |
| **ATCC-7061 vs *B.licheniformis*** | **69** | **63** | **68** | **67** | **68** | **67** | **71** | **71** | **71** |
| **ATCC-7061 vs FO-36b** | **97** | **98** | **97** | **96** | **97** | **96** | **94** | **93** | **93** |
| **FO-36b vs *B.subtilis*** | **70** | **71** | **71** | **76** | **72** | **72** | **73** | **72** | **72** |
| **FO-36b vs *B.licheniformis*** | **70** | **64** | **69** | **66** | **67** | **66** | **73** | **71** | **72** |
| ***B.subtilis* vs *B.licheniformis*** | **70** | **70** | **70** | **71** | **70** | **70** | **67** | **77** | **75** |

*** The *cotVWXYZ* operon encodes spore coat proteins that are critical for sporulation initiation, genetic competence and a stable spore coat [**[**56**](#_ENREF_56)**]. Within this operon, the *cotWX* segment exhibits large sequence variations. In contrast, the SAFR-032 sequences of *cotD*, *cotV*, *cotY* and *cotZ* all have over 93% similarity with the closely related ATCC7061^T^ strain.**

**The rapid changes in the ‘cot’ genes are also seen among other non-resistant *Bacillus* strains. Possibly the “cot” genes are frequently involved in adaptation of *Bacillus* strains to various niches.**

**3 - The *oppABCDF (spo0K)* operon encodes an oligopeptide permease system expressing ATP-binding cassette (ABC) transporters in *B. subtilis* [**[**59**](#_ENREF_59)**].**
