# Supplementary material for: Candidate Genes That May Be Responsible for the Unusual Resistances Exhibited by Bacillus pumilus SAFR-032 Spores
Source: PLoS One. 2013 Jun 14;8(6):e66012. doi: 10.1371/journal.pone.0066012 (PMC3682946; doi:10.1371/journal.pone.0066012)
Supplement: Table S8 — List of DNA repair and peroxide resistance genes and their presence/absence in the three genomes. (DOCX) [file pone.0066012.s014.docx]

**Table S8: List of DNA repair and peroxide resistance genes and their presence / absence in the three genomes**

| **Gene category** | **Name** | **Locus tag no(s)** | | | **Conserved location^3^** | **% Identity** | |
| --- | --- | --- | --- | --- | --- | --- | --- |
|  |  | **SAFR-032 (BPUM no)** | **ATCC-7061**  **(BAT no)** | **FO-36b^2^** |  | **Ω** | **π** |
| **DNA repair** | **DNA (cytosine-5-)-methyltransferase YdiP^1^** | **561** | **1588** | **A** | **no** | **43** | **48-72** |
|  | **Helicase** | **608** | **A** | **A** | **NA** | **NA** | **21-25** |
|  | **ATP-binding protein^1^** | **652** | **A** | **A** | **NA** | **NA** | **37-48** |
|  | **Endonuclease^1^** | **653** | **A** | **A** | **NA** | **NA** | **38-52** |
|  | **DNA (cytosine-5-) methyltransferase** | **656** | **A** | **A** | **NA** | **NA** | **38-43** |
|  | **DNA repair methyltransfrease Ada** | **1200** | **2855** | **P** | **yes** | **90** | **55-62** |
|  | **photolyase PhrB** | **1378** | **2666** | **P** | **yes** | **90** | **44-51** |
|  | **DNA helicase^1^** | **3674** | **A** | **A** | **NA** | **NA** | **30-45** |
| **Peroxide resistance** | **probable dioxygenase** | **0482** | **3585** | **P** | **yes** | **94** | **58-66** |
|  | **TrxB-like thioredoxin-disulfide reductase** | **0664** | **2666** | **P** | **yes** | **89** | **44-52** |
|  | **possible monooxygenase** | **0802** | **3282** | **P** | **yes** | **92** | **28-72** |
|  | **lysine/ornithine N-monooxygenase** | **0931** | **3137** | **P** | **yes** | **90** | **46-60** |
|  | **possible FAD dependent oxidoreductase** | **1153** | **2901** | **P** | **yes** | **88** | **37-38** |
|  | **manganese (Mn) catalase YdbD** | **1305** | **2745** | **P** | **yes** | **93** | **72-86** |
|  | **NADH-dependent flavin oxidoreductase** | **1716** | **A** | **A** | **no** | **NA** | **61-74** |
|  | **flavodoxin^1^** | **1721** | **A** | **P** | **NA** | **91** | **48-53** |
|  | **flavin reductase** | **1731** | **A** | **A** | **NA** | **NA** | **50-83** |
|  | **methionine sulfoxide reductases MsrBA(YppQP) ^1^** | **1900-01** | **A** | **P** | **NA** | **90** | **75-79** |
|  | **NADH dehydrogenase** | **2106** | **1909** | **P** | **yes** | **90** | **57** |
|  | **sporulation related manganese (Mn) catalase (YjqC)** | **2346** | **A** | **P** | **NA** | **89** | **64 -81** |
|  | **Thioredoxin** | **3130** | **A** | **P** | **NA** | **86** | **57-73** |
|  | **Peroxiredoxin** | **3690** | **1135** | **P** | **yes** | **93** | **26-57** |

**P – present; A – absent; -NA- not applicable; 1 - additional genes, not reported in the previous study[10]; 2 – locus tags not available for the draft genome of *B. safensis* F036B and only the presence/absence of the gene(s) indicated; 3 - conserved location (information) on the two *B. pumilus* genomes only; Ω - average % identity of a gene’s homologs from the two closely related JPL isolates *B. pumilus* SAFR-032 and *B. safensis* FO-36b and the *B. pumilus* type strain ATCC-7061; π - average % protein sequence identity with the nearest 10 non *B. pumilus/B. safensis* homologs**
